# MicroRNA-5p and -3p co-expression and cross-targeting in colon cancer cells

**DOI:** 10.1186/s12929-014-0095-x

**Published:** 2014-10-05

**Authors:** Kong Bung Choo, Yuen Loon Soon, Phan Nguyen Nhi Nguyen, Michele Sook Yuin Hiew, Chiu-Jung Huang

**Affiliations:** Department of Preclinical Sciences, Faculty of Medicine and Health Sciences, Centre for Stem Cell Research, Universiti Tunku Abdul Rahman, Selangor, Malaysia; Centre for Stem Cell Research, Universiti Tunku Abdul Rahman, Selangor, Malaysia; Department of Animal Science, Graduate Institute of Biotechnology, School of Agriculture, Chinese Culture University, 55, Hwa-Kang Road, Yang Ming Shan, Taipei 111 Taiwan; Graduate Institute of Biotechnology, School of Agriculture, Chinese Culture University, Taipei, Taiwan

**Keywords:** microRNA-5p and -3p pairs, Colon cancer, miRNA cross targeting, Safe-proof regulation

## Abstract

**Background:**

Two mature miRNA species may be generated from the 5’ and 3’ arms of a pre-miRNA precursor. In most cases, only one species remains while the complementary species is degraded. However, co-existence of miRNA-5p and -3p species is increasingly being reported. In this work, we aimed to systematically investigate co-expression of miRNA-5p/3p in colon cancer cells in a genome-wide analysis, and to examine cross-targeting of the dysregulated miRNAs and 5p/3p species.

**Results:**

Four colon cancer cell lines were examined relative to two normal colon tissues. Of the 1,190 miRNAs analyzed, 92 and 36 were found to be up- or down-regulated, respectively, in cancer cells. Nineteen co-expressed miRNA-5p/3p pairs were further identified suggesting frequent 5p/3p co-accumulation in colon cancer cells. Of these, 14 pairs were co-up-regulated and 3 pairs were co-down-regulated indicating concerted 5p/3p dysregulation. Nine dysregulated miRNA pairs fell into three miRNA gene families, namely *let-7*, *mir-8/200* and *mir-17*, which showed frequent cross-targeting in the metastasis process. Focusing on the let-7d-5p/3p pair, the respectively targeted IGF1R and KRAS were shown to be in a reverse relationship with expression of the respective miRNA, which was confirmed in transient transfection assays using let-7d mimic or inhibitor. Targeting of KRAS by let-7d was previous reported; targeting of IGF1R by let-7d-5p was confirmed in luciferase assays in this study. The findings of let-7d-5p/3p and multiple other miRNAs targeting IGF1R, KRAS and other metastasis-related factors suggest that 5p/3p miRNAs contribute to cross-targeting of multiple cancer-associated factors and processes possibly to evade functional abolishment when any one of the crucial factors are inactivated.

**Conclusions:**

miRNA-5p/3p species are frequently co-expressed and are coordinately regulated in colon cancer cells. In cancer cells, multiple cross-targeting by the miRNAs, including the co-existing 5p/3p species, frequently occurs in an apparent safe-proof scheme of miRNA regulation of important tumorigenesis processes. Further systematic analysis of co-existing miRNA-5p/3p pairs in clinical tissues is important in elucidating 5p/3p contributions to cancer pathogenesis.

**Electronic supplementary material:**

The online version of this article (doi:10.1186/s12929-014-0095-x) contains supplementary material, which is available to authorized users.

## Background

MicroRNAs (miRNAs) are short (~22 nucleotides) single-stranded post-transcriptional regulatory RNAs in major cellular processes. In the canonical miRNA biogenesis pathway, miRNA primary transcripts are processed and exported to the cytoplasm where double-stranded duplexes are generated through the action of Dicer in the RNA-induced silencing complex (RISC) [1,2]. Subsequently, one of the strands, designated as miRNA or the guide strand, is preferentially selected for maturation; the complementary miRNA* strand, or the passenger strand, is destined to be degraded. However, recent reports have indicated that both the miRNA and miRNA* species often co-exist and both are functional [3-7]. The mature miRNA species may be derived from both the 5’ and 3’ arms of the precursor duplex, and are called the miRNA-5p and -3p species, respectively. These findings have introduced some degree of confusion in the miRNA nomenclature. To avoid further confusion and ambiguity by prematurely presuming expression levels and biological functions for a specific miRNA strand, miRBase (http://www.mirbase.org/) has recently retired the human miRNA/miRNA* nomenclature. Instead, the miRNA-5p and -3p nomenclature is now being used based solely on 5’- or 3’-arm derivation of the miRNA species. miRBase further advises authors to always include the miRNA sequences being reported for cross-referencing. In this paper, the 5p/3p nomenclature is used, and the original miR-miR* names are also listed alongside (see Additional file 1).

In a number of deep sequencing studies, co-existence of 5p/3p pairs has been demonstrated in about half of the miRNA populations analyzed and the relative concentrations of the 5p/3p species may be comparable or varied extensively [4,6]. Notably, the minor miRNA species, be they 5p or 3p, are evolutionarily conserved in the seed sequences signifying biological significance [5,7-9]. It is further shown that the relative expression levels of co-existing miRNA pairs vary from tissue to tissue hinting tissue-dependent regulatory roles for the 5p/3p miRNA species [3]. If the miRNA 5p/3p pairs are co-expressed in normal tissues, it is then important to explore how expression of the pairs is altered and regulated in human diseases, particularly in cancers. Indeed, miR-24-2, miR-146, miR-28 and miR-125a and miR-17, to name just a few, have been shown to be co-expressed in pairs in breast, thyroid, colorectal, lung and liver cancers, respectively [10-14]. Besides cancers, paired species of members of the *let-7* and *mir-126* families also co-exist and shown to play different roles in regulating reprogramming and differentiation in embryonic stem cells [15,16].

Despite reports on the involvement of specific miRNAs in cancers, genome-wide studies focusing on the participation of miRNA-5p/3p pairs in the tumorigenesis processes are still lacking. This work aimed to systematically investigate co-expression and regulation of 5p/3p paired miRNA species in cancer cells. Colon cancer, the third most prevalent cancer worldwide [17], was used as a study model. Although many papers have been published on miRNA profiling in colon cancer using different microarray platforms [18-21], none has compared 5p/3p contributions. In this work, a nanolitre-scale real-time reverse transcription-PCR (qRT-PCR) platform was used for differential miRNA profiling in colon cancer cells relative to normal colon tissues. Our data indicate that miRNA 5p/3p pairs are frequently co-expressed and co-regulated in colon cancer cells. Furthermore, the dysregulated miRNAs and 5p/3p pairs are frequently involved in cross-regulation of multiple targets in pathways in the tumorigenesis process.

## Methods

### Colon cancer cell lines and normal colon tissues

Four human colon cancer cell lines, HCT-15, HT-29, SK-CO-1 and WiDr (ATCC, Manassas, VA) and total RNA samples isolated from two independent sources of non-cancerous colon tissues (Origene, Rockville, MD) were used in this work.

### Nomenclature

Throughout this work, the miRNA-5p/-3p nomenclature as recommended by miRBase, Release 19, was used. For cross referencing, a list of the 5p/3p designations, the miRNA sequences and the previous names of miRNA/miRNA* is shown in Additional file 1. All miRNAs described in this work are human miRNA. For simplicity, the *hsa-* prefix has been dropped from all miRNA designations in the text.

### RNA preparation, microarray processing and data analyses

Total RNAs were isolated from the colon cancer cells or normal tissues using the RNeasy Plus Mini Kit (Qiagen, Valencia, CA) according to the manufacturer’s instructions. One microgram RNA was applied to a SmartChip Human MicroRNA Chip, Panel v2 (WaferGen Biosystems, Fremont, CA), for high-throughput nanolitre-scale qRT-PCR microarray analysis as described previously [22]. It is noteworthy that at the time of writing, there were only 261 5p/3p miRNA pairs (522 miRNAs) included in the profiling panel. The assays were performed in quadruplicates and included eleven endogenous and six exogenous data quality controls. The data obtained with the colon cancer cell lines were normalized to those of the normal colon tissues. Data were analyzed using the comparative cycle threshold (ΔΔC_т_) method and statistical analysis.

### MicroRNA and mRNA quantitative real-time RT-PCR

Real-time qRT-PCR was performed using the NCode SYBR GreenER miRNA qRT-PCR kit (Invitrogen, Carlsbad, CA) following the supplier’s instructions in a Rotor-Gene Q real-time PCR cycler (Qiagen). Following miRNA poly(A) tailing, first-strand cDNA was synthesized using the Superscript III RT/RNaseOUT enzyme mix provided in the kit, followed by real-time RT-PCR using SYBR® GreenER™ qPCR SuperMix Universal (Invitrogen) in Rotor-Gene Q for UDG incubation at 50°C for 2 min and UDG inactivation and DNA polymerase activation at 95°C for 10 min. Amplification was carried out for 40 cycles at 95°C for 15 sec and primer annealing at 58°C for 1 min. Experiments were performed in triplicates and were normalized to the data of the small nuclear RNA (snRNA) *U6*. Primers used for miRNA quantification are as in Additional file 1. The U6 oligonucleotide 5’-CACCACGTTTATACGCCGGTG-3’ was used as the normalization control. Relative miRNA levels were calculated using the comparative ΔΔC_т_ method. Similarly, mRNA was quantified by real-time RT-PCR using SYBR® GreenER™ qPCR SuperMix Universal (Invitrogen) at the same RT-PCR condition as above, except for annealing temperature of primers at 60°C for 1 min. Experiments were performed in triplicates and were normalized to the data of *GAPDH*. Primers used in RT-PCR of mRNAs were as follows: IGF1R-F: 5’-CAAGGCCTGAAAACTCCATC-3’ and IGF1R-R: 5’-CGCTGATCCTCAACTTGTGA-3’; KRAS-F: 5’-TGAGGACTGGGGAGGGCTTTCTT-3’ and KRAS-R: 5’-AGAAGGCATCATCAACACCCTGTCT-3’; GAPDH-F: 5’-GAAATCCCATCACCATCTTCCAGG-3’, GAPDH-R: 5’-GAGCCCCAGCCTTCTCCATG-3’. Relative mRNA levels were calculated using the comparative ΔΔC_т_ method.

### miRNA stem-loop RT-PCR

Detection of miRNA by stem-loop RT-PCR was as described previously [22]. For cDNA synthesis, the annealing of the stem-loop primers (Additional file 2) was 5 min at 65°C. The stem-loop products were then used for reverse transcription using Superscript III reverse transcriptase (Invitrogen) as previously described [22] followed by PCR amplification using the following incubation conditions: 98°C for 5 min, followed by 40 cycles of 98°C for 10 s, 60°C for 30 s, 72°C for 1 min. The reaction was terminated after a further 70°C extension for 10 min. In the stem-loop PCR, U6 was included as an internal control. The PCR products were displayed by electrophoresis on a 4% agarose gel.

### Preparation of cell lysates and western blot analysis

Cells were washed twice with ice cold phosphate-buffered saline, and harvested by lysis in TGH buffer (1% Triton X-100; 10% glycerol; 50 mM HEPES, pH 7.3; 1% deoxycholic acid; 10 mM N-ethylmaleimide; 5 mM EDTA; 1 mM EGTA and protease inhibitors, 10 mM NaF, 1 mM sodium orthovanadate, 1 mM DTT, 2 mM phenylmethylsulfonyl fluoride, 10 μg/ml aprotinin, 10 μg/ml leupeptin). Aliquots of 35 μg protein per lane were separated on 10% SDS-PAGE, semi-dry transferred to a nitrocellulose membrane and blotted with monoclonal antibodies against IGF1R (05–656, Upstate, Merck KGaA, Darmstadt, Germany) or KRAS (05–516, Upstate) and using an monoclonal antibody against GAPDH (ab8245, Abcam, Cambridge, UK) as a loading control, followed by horseradish–peroxidase (HRP)-conjugated secondary antibody. Visualization was achieved by using chemiluminescence (Amersham ECL, Freiburg, Germany). Band densitometric analysis was performed by VisionWorks®LS Image Acquisition and Analysis Software (Ultra-Violet Products, Cambridge, UK).

### Transient transfection with miRNA mimic or inhibitor

HCT-15 cells were seeded onto 6-well plate at a density of 1 × 10^6^ cells/well and were transiently transfected with 30 nM mirVana miRNA let-7d-5p inhibitor, or let-7d-3p mimic, or the appropriate miRNA inhibitor or mimic negative control (Applied Biosystems, Foster City, CA) using Lipofectamine RNAiMAX Reagent (Invitrogen). Forty-eight hours post-transfection, the transfected cells were harvested for miRNA, mRNA and protein assays.

### Plasmid construction and site-directed mutagenesis

Sequences harboring each of the three putative let-7d-5p targeted sites were generated by PCR using specific primers (Additional file 3) for cloning into the XbaI site of the pGL3-Control vector (Promega, Madison, WI). The cloned 3’-UTR segments of IGF1R were as follows: IGF1R-1, nucleotide (nt) 4,169-4,344 (176 bp); IGF1R-2, nt. 6,698-6,930 (233 bp); IGF1R-3, 10,697-10.897 (201 bp), using the accession number NM_000875 IGF1R mRNA sequence as the reference. Mutations of the miRNA seed sequences were performed using primers shown in Additional file 3 and using the QuikChange® Lightning Site-Directed Mutagenesis kit (Agilent Technologies, Santa Clara, CA) as recommended by the supplier. The mutations were confirmed by sequencing.

### Transient transfection and luciferase assays

Transient transfection into HCT-15 cells was performed in triplicates using the PLUS™ Reagent and Lipofectamine 2000™ (Invitrogen) as previously described [23,24]. A validated let-7d-5p mimic and the mimic negative control (Ambion, Foster City, CA) were used in co-transfection. Luciferase assays were performed 48 h post-transfection using the Dual-Luciferase Reporter 1000 Assay kit (Promega) [23,24].

### Bioinformatics analyses for target prediction and gene ontology

Hierarchical clustering and volcano plot analyses were performed using miScript miRNA PCR Array Data Analysis Web Portal. miRNA target transcripts were predicted using web-based algorithms, including miRBase (www.mirbase.org), TargetScan Release 6.2 (http://www.targetscan.org/) TarBase (http://diana.cslab.ece.ntua.gr/), miRTarBase (http://mirtarbase.mbc.nctu.edu.tw/), microRNA.org (www.microrna.org) and miRDB (www.mirdb.org). To filter the large number of predicted targets, criteria and parameters were set for seed-sequence matching, conservation of miRNA binding sites in the targeted mRNA sequences across species and thermostability of the miRNA:target duplexes. Gene ontology analysis was performed to identify the functions of the predicted targets by using the Gene Ontology Annotation-UniProt-GOA database (http://www.ebi.ac.uk/GOA/) and the KEGG pathway database (www.genome.jp/kegg/pathway.html).

### Statistical analysis

Statistical analysis was performed using the SPSS software for Windows v11.5. Results are described as averages of log_2_ (fold change) ± standard deviation (SD). Data were analyzed by paired Student’s *t*-test (two-tailed distribution) comparing the differences of miRNA levels between colon cancer cells and normal colon tissues. Statistical significance was accepted at *p* < 0.05.

## Results

### Co-expression and concerted dysregulation of miRNA-5p/3p pairs

**Figure 1:**
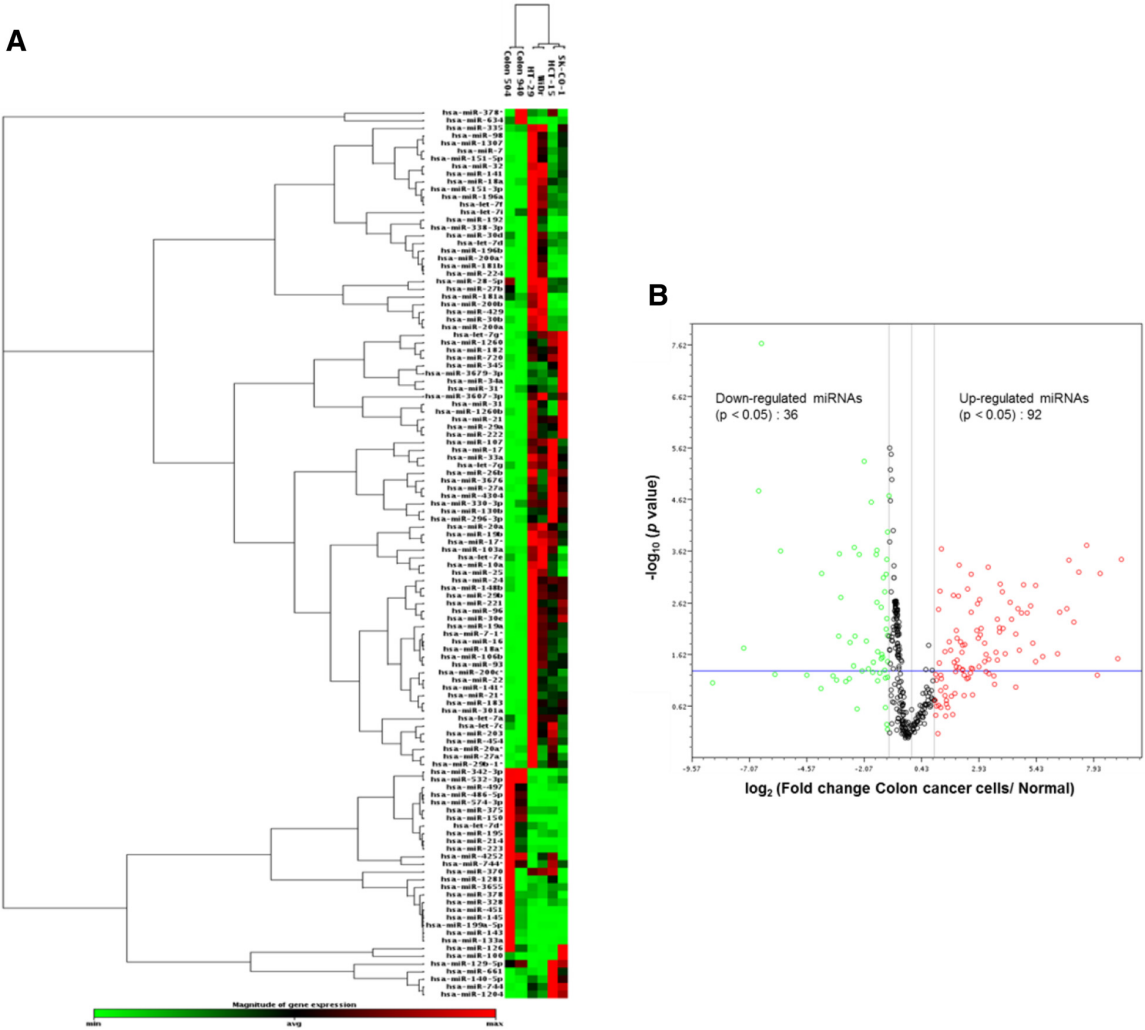
**MicroRNA profiling of colon cancer cells. (A)** Hierarchical Clustering Analysis of differentially regulated miRNAs in the HCT-15, HT-29, SK-CO-1 and WiDr colon cancer cells as compared to cells of two normal colon tissues. **(B)** Volcano plot analysis. Green circles indicate minimum level of miRNA expression (log_2_FC < −1.5), black circles indicate average or weak miRNA expression, red circles indicate strong or maximum level of miRNA expression (log_2_FC > 1.5). FC: fold change.

To systematically investigate co-expression of miRNA-5p and -3p pairs in colon cancer cells, global miRNA profiling of four colon cancer cell lines, HCT-15, HT-29, SK-CO-1 and WiDr, and two normal colon tissues were first performed using a nanolitre-scale real-time RT-PCR microarray platform which included 1,190 human miRNAs. Hierarchical clustering analysis demonstrated separation of normal from cancer cells although the separation was incomplete (Figure 1A). Furthermore, the four colon cancer cell lines were found in two sub-clusters: sub-cluster 1 was represented by HT-29 and WiDr cells and sub-cluster 2 included HCT-15 and SK-CO-1 cells. Interestingly, HT-29 and WiDr cells were originally derived from low and non-metastatic colon cancers, whereas HCT-15 and SK-CO-1 cells were derived from high metastatic or high invasive potential colon cancer [25,26]. This observation suggests possible association of unique miRNA expression profiles with the metastatic and non-metastatic nature of colon cancer cells. Volcano plot analysis was further performed to sort out the miRNAs with statistically significant differential expression (*p* < 0.05) and log_2_ (fold change) values with cut-off point set at ±1.5 (Figure 1B). Amongst the differentially expressed miRNAs, 92 (71.9%) were significantly up-regulated and 36 (28.1%) were down-regulated, suggesting significant miRNA dysregulation in colon carcinogenesis, which is consistent with previous reports [18-21].

**Figure 2:**
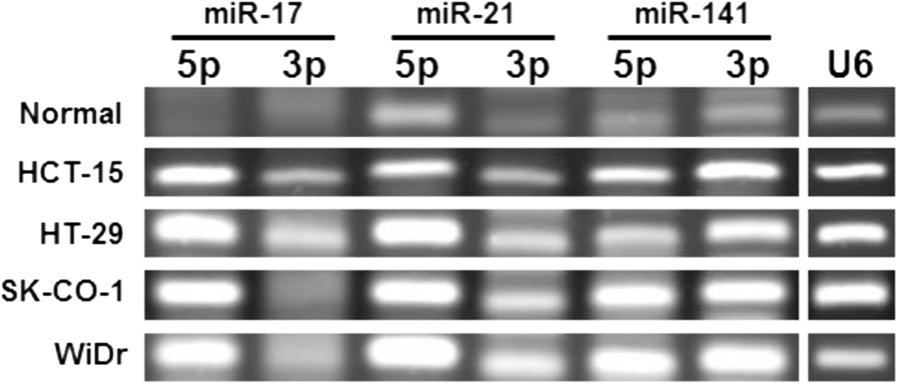
**Co-expression of miRNA-5p and -3p pairs in colon cancer cells.** miRNA expression was determined by stem-loop RT-PCR using U6 snRNA as a PCR control. The PCR products were analyzed in 4% agarose gels.

To focus on co-expression of the 5p/3p miRNAs, data of 5p/3p pairs, with at least one of the pair with log_2_(fold change) ≥ 1.5 or ≤ −1.5, were extracted from the dataset of the 128 dysregulated miRNAs. A total of 19 pairs (38 miRNAs) that answered to this criterion were obtained (Table 1). The alterations observed in the cancer cells relative to the normal tissues ranged from significant up-regulation by 9.13-fold in miR-21-5p to down-regulation by 3.94-fold in miR-574-3p. Out of the 19 miRNA pairs thus extracted, it is further observed that 14 (73.7%) 5p/3p pairs were co-up-regulated and 3 (15.8%) pairs were co-down-regulated. Only 2 (10.5%) pairs, miR-200b and let-7d, showed reverse directions of dysregulation in the 5p/3p species. Co-expression was validated in three randomly selected miRNA pairs, miR-17, -21, -141, in the co-up-regulated group by stem-loop RT-PCR in all four cervical cancer cell lines and in a normal colon tissue (Figure 2). Up-regulated expression of the three miRNA pairs was evident when the expression levels in the cancer cell lines were compared with the normal tissues. Furthermore, co-expression of the 5p/3p pairs was also shown for miR-21 and -141. However, In the case of miR-17, however, despite the observation that the 3p levels in SK-CO-1 and WiDr were much lower than the 3p species, 5p/3p co-expression was clearly shown for HCT-15 and HT-29. To validate co-regulation, one miRNA pair was randomly chosen from each of the three categories of regulation in Table 1 for direct real-time quantitative RT-PCR assays (Table 2). The data obtained were in excellent agreement with the microarray data, thus supporting the validity of the miRNA profiling data presented. Reverse dysregulation of the let-7d-5p/3p pair was also confirmed. Taken together, the data show frequent co-expression of 5p/3p miRNAs in colon cancer cells, and that the majority (89.5%) of the co-expressed 5p/3p pairs was co-up- or co-down-regulated, strongly suggesting concerted dysregulation of miRNA sister pairs in colon cancer cells.

**Table 1:** **Altered expression of miRNA-5p and -3p pairs in colon cancer cells relative to normal cells** ^**1**^

**Family**	**miRNA-5p**	**Log**_**2**_ **(fold change)**	**miRNA-3p**	**Log**_**2**_ **(fold change)**
		**(Mean ± SD)**		**(Mean ± SD)**
**1. 5p/3p co-upregulated (n = 14)**				
*let-7*	let-7g-5p	2.97 ± 0.49**	let-7g-3p	2.12 ± 0.13*
*let-7*	let-7i-5p	2.81 ± 1.27*	let-7i-3p	1.67±0.45*
*mir-7*	miR-7-5p	6.44 ± 1.14*	miR-7-1-3p	3.83±0.66**
*mir-8/200*	miR-141-5p	3.85 ± 0.76**	miR-141-3p	4.50 ± 1.08**
*mir-8/200*	miR-200a-5p	5.17 ± 0.65**	miR-200a-3p	8.10 ± 1.58**
*mir-17*	miR-17-5p	6.68 ± 0.69**	miR-17-3p	4.12 ± 0.59**
*mir-17*	miR-18a-5p	3.94 ± 0.91**	miR-18a-3p	3.18 ± 0.77**
*mir-17*	miR-20a-5p	7.19 ± 0.78**	miR-20a-3p	1.58 ± 0.98
*mir-21*	miR-21-5p	9.13 ± 0.39**	miR-21-3p	5.99 ± 0.85**
*mir-22*	miR-22-5p	1.98 ± 0.78	miR-22-3p	3.22 ± 0.76**
*mir-27*	miR-27a-5p	2.94 ± 1.21**	miR-27a-3p	4.78 ± 0.62**
*mir-28*	miR-151a-5p	2.65 ± 0.67*	miR-151a-3p	5.39 ± 1.20 **
*mir-29*	miR-29b-1-5p	0.93 ± 0.84	miR-29b-1-3p	7.62 ± 0.32**
*mir-31*	miR-31-5p	8.88 ± 1.73**	miR-31-3p	2.08 ± 0.76**
**2. 5p/3p co-downregulated (n = 3)**				
*mir-199*	miR-199a-5p	−3.84 ± 2.16**	miR-199a-3p	−3.23 ± 1.55**
*mir-378*	miR-378a-5p	−2.60 ± 0.008**	miR-378a-3p	−1.04 ± 0.27
*mir-574*	miR-574-5p	−2.90 ± 0.44*	miR-574-3p	−3.94 ± 0.21**
**3. 5p/3p inversed regulation (n = 2)**				
*let-7*	let-7d-5p	2.29 ± 1.01	let-7d-3p	−2.78 ± 0.79**
*mir-8/200*	miR-200b-5p	−1.67 ± 0.39	miR-200b-3p	3.15 ± 0.25**

^1^Based on miRBase 19 data; a full list of alternative miRNA names is provided in Additional file 1. *p < 0.0 5, **p < 0.01.

**Table 2:** **Validation of microarray data of selected miRNA-5p and -3p pairs by real-time qRT-PCR**

**miRNA-5p**	**Log**_**2**_ **(fold change)**		**miRNA-3p**	**Log**_**2**_ **(fold change)**	
	**(Mean ± SD)**			**(Mean ± SD)**	
	**WGBS**	**qRT-PCR**		**WGBS**	**qRT-PCR**
miR-20a-5p	7.19 ± 0.78**	7.74 ± 1.17**	miR-20a-3p	1.58 ± 0.98	4.23 ± 1.01**
miR-378a-5p	**−**2.60 ± 0.01**	**−**1.13 ± 0.87	miR-378a-3p	**−**1.04 ± 0.27	**−**1.06 ± 1.01
let-7d-5p	2.29 ± 1.01	4.08 ± 0.87**	let-7d-3p	**−**2.78 ± 0.79**	**−**2.96 ± 0.33**

The mean ± standard deviation (SD) real-time quantitative RT-PCR (qRT-PCR) data were obtained in three independent experiments on all four colon cancer cell lines as described in the text. WGBS, data from the WaferGen Biosystems microarray analysis. **p < 0.01.

### Cross-targeting of dysregulated 5p/3p miRNAs on metastasis-related functions in colon cancer cells

Nine of the 19 dysregulated miRNA pairs fell into 3 different miRNA families, namely *let-7, mir-8/200* and *mir-17*. Transcripts targeted by the 5p and 3p members of the three families were sourced from the miRBase database, Release 19 (Table 3; see also Additional file 4). When the number of predicted targets was examined, it is noted that out of the 9 pairs analyzed, 7 pairs (77.78%) showed approximately equal numbers of predicted target mRNAs for both the 5p and 3p species indicating little or no bias in arm selection, a hint that the co-expressed 5p and 3p miRNAs may both be functionally significant [3]. The two exceptions were let-7d and -7i which showed approximately 4:1 ratio in the number of predicted targets for the 5p/3p species. Deviations from an equal ratio for the relative concentrations of the 5p/3p species suggest uneven evolutionary pressures on arm selection for let-7d and -7i, echoing the findings of Griffiths-Jones *et al.* [5]. The mechanism and the biological significance of unbiased or preferred arm selection remain to be elucidated.

**Table 3:** **Distribution of predicted miRNA-5p and -3p target transcripts**

**miRNA**	**5p/3p distribution**
**(a)** ***let-7*** **family**	
let-7 g-5p	0.446
let-7 g-3p	0.554
let-7d-5p	0.809
let-7d-3p	0.191
let-7i-5p	0.816
let-7i-3p	0.184
**(b)** ***mir-8/200*** **family**	
miR-200a-5p	0.485
miR-200a-3p	0.515
miR-200b-5p	0.504
miR-200b-3p	0.496
miR-141-5p	0.485
miR-141-3p	0.515
**(c)** ***mir-17*** **family**	
miR-17-5p	0.496
miR-17-3p	0.504
miR-18a-5p	0.476
miR-18a-3p	0.524
miR-20a-5p	0.554
miR-20a-3p	0.466

For full details, see Additional file 4.

Involvement of miRNAs in the metastasis processes has previously been demonstrated in human cancers (reviewed in [27]). As a study model in the present work, targeted transcripts and the predicted biological functions of the 19 dysregulated miRNA pairs were derived *in silico* (see Additional file 5). For clarity in analysis, only two better-characterized targets for the 5p/3p members of the three miRNA families in Table 3 were selected for further dissertation in this work (Table 4).

**Table 4:** **Metastasis-associated biological functions of selected target transcripts of miRNA-5p and -3p sister pairs** ^**1**^

**miRNA family**	**miRNA**	**Chrom’l location**	**Expression**	**Selected target mRNAs** ^**2**^	**Functions** ^**3**^
*let-7*	let-7 g-5p	3p21.1	Up-reg’d	IGF1R*	Angiogenesis, apoptosis
				MYC*	Angiogenesis, apoptosis, cancer, Jak-STAT, MAPK & Wnt signaling
	let-7 g-3p		Up-reg’d	ZEB1	EMT, transcriptional misregulation in cancer
				NLK	Angiogenesis, MAPK & Wnt signaling
	let-7d-5p	9q22.32	Up-reg’d	IGF1R*	Angiogenesis, apoptosis
				THBS1*	Angiogenesis, cell cycle, p53 signaling, cancer
	let-7d-3p		Down-reg’d	KRAS	Apoptosis, MAPK & VEGF signaling, cancer
				PRKACB	Apoptosis, MAPK, Wnt & Hedgehog signaling
	let-7i-5p	12q14.1	Up-reg’d	TLR4*	Toll-like receptor signaling, inflammatory response
				IL13*	Cell-cell signaling, Jak-STAT, Toll-like receptor signaling, cytokine-cytokine receptor interaction
	let-7i-3p		Up-reg’d	DLX5	Cell proliferation
				ACTB	Focal adhesion, leukocyte transendothelial migration
*mir-8/200*	miR-200a-5p	1p36.33	Up-reg’d	ZEB2	EMT, Wnt signaling
				FGF4	Apoptosis, MAPK signaling
	miR-200a-3p		Up-reg’d	ZEB1*	EMT, transcriptional misregulation in cancer
				ZEB2*	EMT, Wnt signaling
	miR-200b-5p	1p36.33	Down-reg’d	ZEB2	EMT, Wnt signaling
				PRDM6	Chromatin modification
	miR-200b-3p		Up-reg’d	ZEB1*	EMT, transcriptional misregulation in cancer
				MLH1	Mismatch repair, cancer
	miR-141-5p	12p13.31	Up-reg’d	ATM	Apoptosis, cell cycle, p53 signaling, cancer
				CLDN1	Cell adhesion, leukocyte transendothelial migration
	miR-141-3p		Up-reg’d	MYC*	Angiogenesis, apoptosis, cancer, Jak-Stat, MAPK & Wnt signaling
				TP53*	Angiogenesis, apoptosis**,** cell cycle, MAPK, p53 & Wnt signaling, cancer
*mir-17*	miR-17-5p	13q31.3	Up-reg’d	THBS1*	Angiogenesis, cell cycle, p53 signaling, cancer
				E2F1*	Apoptosis, cell cycle, cancer
	miR-17-3p		Up-reg’d	CD44	Angiogenesis, inflammation, cancer
				MYB	Cancer
	miR-18a-5p	13q31.3	Up-reg’d	THBS1*	Angiogenesis, cell cycle, p53 signaling, cancer
				HIF1A*	Angiogenesis, cell migration, VEGF signaling
	miR-18a-3p		Up-reg’d	KRAS*	Apoptosis, MAPK & VEGF signaling, cancer
				CASP7	Apoptosis
	miR-20a-5p	13q31.3	Up-reg’d	THBS1*	Angiogenesis, cell cycle, p53 signaling, cancer
				E2F1*	Apoptosis, cell cycle, cancer
	miR-20a-3p		Up-reg’d	KRAS	Apoptosis, MAPK & VEGF signaling, cancer
				PCNA	Cell cycle, cancer

^1^A complete list of predicated/validated target transcripts and putative regulated biological functions is presented in Additional file 5. ^2^Asterisks in the column indicate validated targets as tabulated in miRBase. ^3^Data were derived from the Gene Ontology Annotation Database (UniProt-GOA) and the KEGG Pathway databases.

On close examination of the putative metastasis-associated functions of the miRNA targets (Table 4), many targeted transcripts are noted to translate for proteins that are engaged in epithelial-mesenchymal transition (EMT), angiogenesis, apoptosis, cell cycle and various signaling pathways. However, some 5p/3p pairs appear to target transcripts that translate proteins of related functions. This is best reflected in miR-200a and -200b in which both the 5p and 3p species regulate transcripts of the metastasis-associated ZEB1 and/or ZEB2 (zinc finger E-box binding homeobox 1 and 2) proteins [28,29] (Table 4). ZEB1 is also the predicted target of let-7g-3p. Regulation of the same transcript by sister pairs or by different miRNA families is probably achieved through recognition of different binding sites of the same target transcript [3,30]. In the *let-7* family, IGF1R (insulin-like growth factor 1 receptor) is the validated target of let-7g-5p and let-7d-5p and regulates angiogenesis and apoptosis All 5p members of *mir-17* have been validated to target THBS1 (thrombospondin 1), which regulates the TP53 pathway, possibly as a result of significant sequence homology within the seed sequences of the family members [31]. Besides *mir-17*, THBS1 is also targeted by the *let-*7 family. Hence, our data demonstrate that both the co-expressed the 5p/3p miRNAs often cross-regulate multiple targets of similar functions in cancer cells. On the other hand, the same target may be regulated by multiple miRNA families: KRAS is simultaneously targeted by members of let-7 and mir-17, ZEB1/2 by let-7 and mir-8/-200 and THBS1 by let-7 and mir-17 families, respectively. It is noteworthy that KRAS is targeted by only the 3p miRNAs whereas THBS1 is the target of 5p species of the *mir-17* family (Table 4). Taken together, our data indicate that 5p and/or 3p miRNAs of different families may target at the same transcript, or a miRNA may cross-regulate different target transcripts translating for proteins with related functions in the metastasis processes. Such cross-targeting suggests a fail-proof mode of miRNA regulation to ensure survival of the cancer cell phenotype.

### Let-7d-5p and -3p target IGF1R and KRAS, respectively

**Figure 3:**
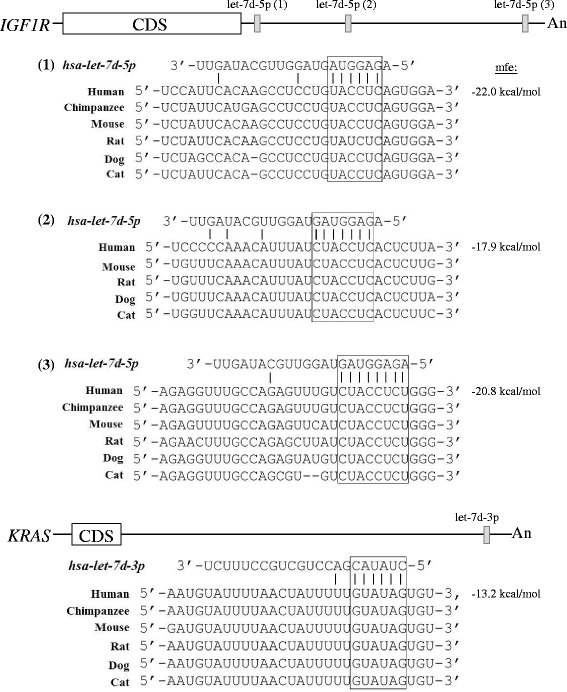
**Targeting of let-7d-5p and -3p at the IGF1R and KRAS mRNAs, respectively.** The *IGF1R* and *KRAS* mRNAs are depicted showing the coding sequence (CDS), the 5’- and 3’-UTR (horizontal lines) and the polyA sequence (An). The three let-7d-5p target sites in the 3’-UTR of *IGF1R* transcript and the single let-7d-3p site in the *KRAS* mRNA are shown by solid vertical bars. The seed sequences of the miRNA alignment with the target transcripts are boxed. mfe, minimal free energy.

**Figure 4:**
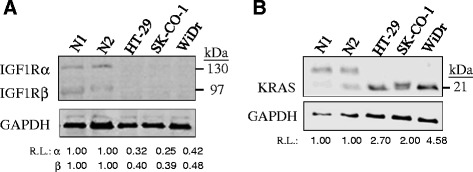
**IGF1R (A) and KRAS (B) protein levels in colon cancer cells.** In the western blot analysis, three colon cancer cell lines, WiDr, SK-CO-1 and HT-29 and two normal colon tissues, N1 and N2, were used. R.L., relative levels after normalizing to 1.00 in the normal samples. In KRAS, the nature of the upper spurious band was unknown. R.L. of KRAS was calculated by taking the mean intensity of the two normal bands as 1.00.

IGF1R and KRAS targeting by let-7d-5p and 3p was further experimentally validated since the two miRNA species were differentially expressed in colon cancer cells in reverse directions: let-7d-5p was up-regulated whereas let-7d-3p was down-regulated (see Tables 1 and 2). Hafner *et al.* [32] previously demonstrated let-7d regulation of IGF1R but which of the 5p/3p species was involved was not specified. Bioinformatics interrogation has revealed putative let-7d-5p targeting of the 7,088-bp 3’-untranslated region (3’-UTR) of IGF1R mRNA at three different locations (Figure 3). Likewise, let-7d-3p is predicted to target at a unique site at the 3’-terminus of the 4,549-bp 3’-UTR of the KRAS mRNA (Figure 3). The putative miRNA sites of the two mRNAs are highly conserved in mammals (Figure 3). To examine post-transcriptional regulation, the IGF1R and KRAS protein levels were first examined in the colon cancer cell line and in normal colon tissues (Figure 4). Results showed that the IGF1R-α and -β subunits, which are cleavage product of the same IGF1R precursor, were 0.33- and 0.53-fold, respectively, of the levels in normal cells, and both were significantly down-regulated in cancer cells (Table 5). On the other hand, the KRAS protein was significantly up-regulated in cancer cells by up to 3.57-fold despite down-regulated mRNA levels (Figure 4 and Table 5). A spurious band which has never been described in the literature appeared in the KRAS western blot of the normal colon cancer tissue. The band was likely a KRAS isoform, but the exact nature was not further determined.

**Table 5:** **Expression levels of let-7d-5p and -3p and associated putative target transcripts and proteins in colon cancer cell lines**

**miRNA**	**miRNA expression** ^**1**^	**Target transcript**	**Protein expression** ^**2**^
	**(Mean ± SD)**		**(Relative to normal)**
let-7d-5p	2.29 ± 1.01	IGF1R	0.33 ± 0.23** (α)
			0.53 ± 0.37** (β)
let-7d-3p	−2.78 ± 0.79**	KRAS	3.57 ± 1.99**

The colon cancer cells lines used were HT-29, SK-CO-1 and WiDr. ^1^Real-time PCR was used to determine miRNA and target mRNA levels; the log_2_ (fold change) values are shown. ^2^Western blotting was used to determine protein levels and the values were expressed relative to those of normal tissues. In both real-time PCR and western analysis, data were derived from three independent experiments. **p < 0.01.

**Figure 5:**
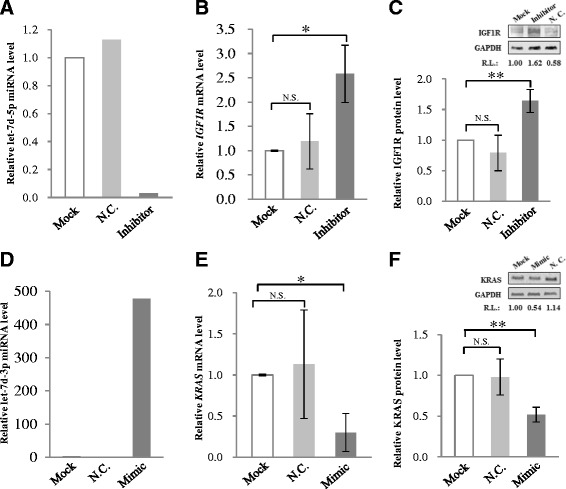
**Effects of altered endogenous let-7d-5p and -3p levels on IGF1R and KRAS expression.** In the experiments, a let-7d-5p inhibitor **(A-C)** or a let-7d-3p mimic **(D-F)** was transfected to HCT-15 cells for 48 h before further assays. The let-7d-5p or -3p miRNA **(A & D)** and the *IGF1R* and *KRAS* mRNA levels **(B & E)** were determined by real-time PCR; the IGF1R and KRAS protein levels **(C & F)** were determined by western blot analysis. The data shown were derived from three independent experiments. In **(C)** & **(F)**, a representative western blot of IGF1R or KRAS is also shown. N.C., a validated negative control; R.L., relative levels compared with the mock control. *p < 0.05, **p < 0.01 and N.S. indicates statistically not significant relative to the mock control.

To further validate let-7d-5p/3p targeting at IFG1R and KRAS, effects of altering endogenous miRNA levels on the target mRNAs and proteins were investigated. When the endogenous let-7d-5p level was knockdown by transfection of a specific let-7d-5p inhibitor sequence in HCT-15 cells (Figure 5A), *IGF1R* mRNA was significantly up-regulated by 2.58 ± 0.59-fold whereas transfection of a negative control with a scrambled sequence had insignificant effects on the *IGF1R* mRNA levels (Figure 5B). On the other hand, the IGF1R protein level was significantly up-regulated by 1.64-fold relative to the mock control (Figure 5C). Hence, knocking down let-7d-5p had clearly led to up-regulation of IGF1R, consistent with let-7d-5p regulation of IGF1R. When a let-7d-3p-specific mimic sequence was transfected into HCT-15 cells, a 478-fold increase of the let-7d-3p level was achieved (Figure 5D). The increased level of the miRNA was accompanied by significant down-regulation of the *KRAS* mRNA level to 0.30-fold (Figure 5E), and also significant down-regulation of the KRAS protein to 0.52-fold that of the mock control (Figure 5F), supporting let-7d-3p regulation of KRAS apparently via increased degradation of the KRAS transcript.

**Figure 6:**
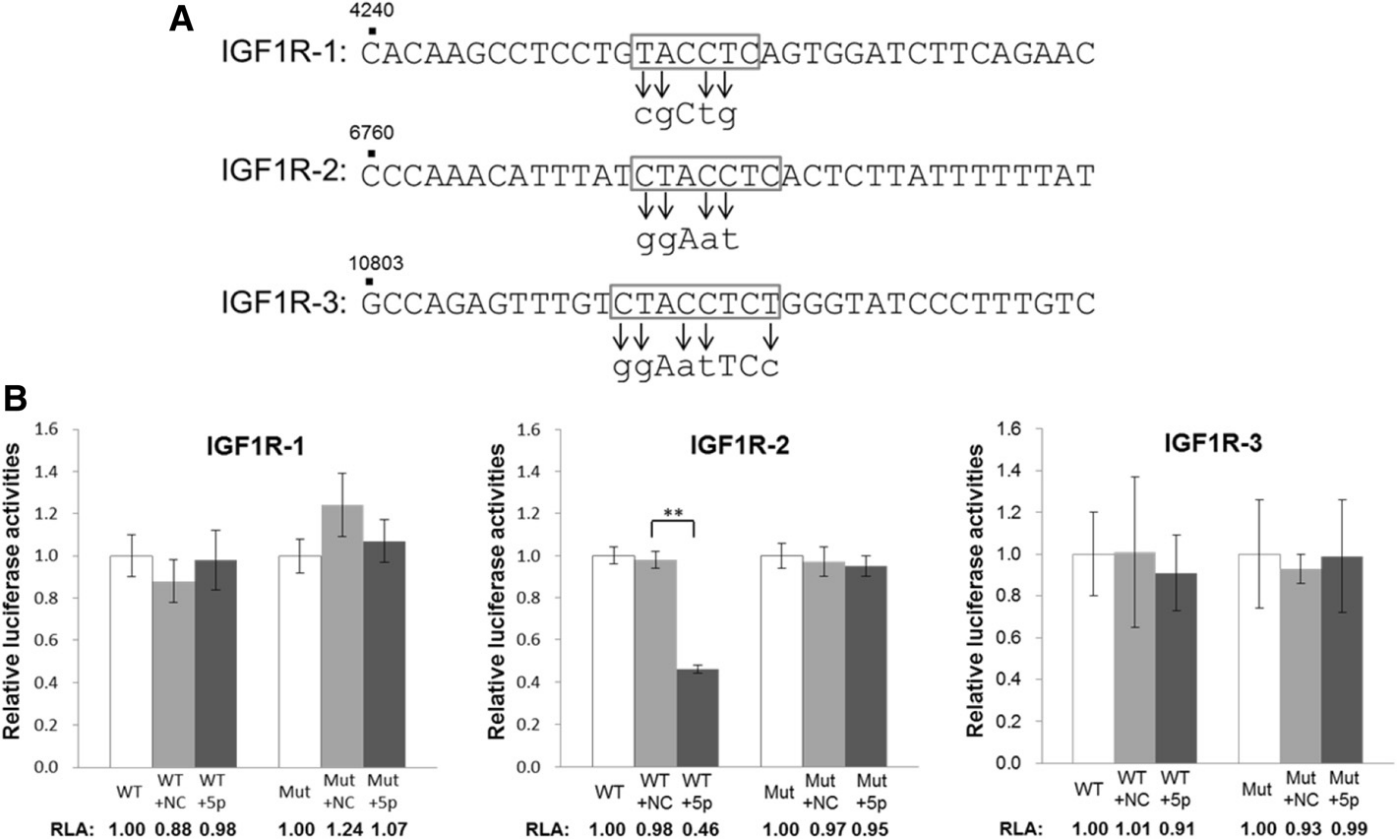
**Targeting of IGF1R by let-7d-5p as shown in luciferase assays. (A)** The seed sequences (boxed) of the three putative let-7d-5p-targeted sequences in IGF1R (see also Figure 3 for the designations and locations of IGF1R-1, -2 and -3). The mutated nucleotides are indicated by arrows and lowercase letters. The nucleotide positions of the sequences harboring the targeted site are shown above the sequences in reference to NM_000875. **(B)** Luciferase assays of transfection of the wild-type (WT) and mutant (Mut) luciferase constructs alone, or in the presence of a let-7d-5p mimic (5p) or a negative control (NC). Relative luciferase activity (RLA) values were calculated relative to the wild-type or mutant construct only, which were arbitrarily set as 1.0. The data were obtained from three independent experiments. **p < 0.001.

Targeting of KRAS by let-7d has been confirmed in a number of previous studies although the 5p or 3p species was not specified [33,34]. Our analysis clearly supports that it is the let-7d-3p that is targeting KRAS (Figure 3). On the other hand, further experimental evidences were needed to support let-7d-5p targeting IGF1R, and to determine which, or if all, of the three predicted target sites in the 3’-UTR of the IGF1R mRNA (Figure 3) is targeted. To achieve this goal, luciferase assays were performed using the pGL-3-Control vector in which about 200-bp oligonucleotides harboring each of the three let-7d-5p targeting sites in the IGF1R sequence (see Methods, and Additional file 3) were inserted at the 3’-end of the luciferase gene of the vector as previously described [24]. Mutants in the seed sequences of the miRNA target sites were also created (Figure 6A). The wild-type and the mutant constructs were transfected into HCT-15 cells alone, or in the presence of a let-7d-5p mimic for miRNA over-expression, or a negative control (NC) oligonucleotide (Figure 6B). The results showed that on over-expressing let-7d-5p, the presence of the second predicted let-7d-5p site, designated as IGF1R-2, inserted in the luciferase vector clearly led to significant down-regulated luciferase activities to 46% of the wild-type level; on the other hand, when the IGF1R-2 site was mutated, no appreciably effects on the luciferase activities were observed (Figure 6B, middle panel). IGF1R-1 and -3 sites were apparently not targeted by let-7d-5p, although the IGF1R-3 site showed a 10% decrease in luciferase activities. Our collective data confirmed that let-7d-5p targeted IGF1R at one of the three predicted sites to result in down-regulated IGF1R expression in colon cancer cells.

## Discussion

MicroRNA involvement in the pathogenesis of human cancers has been well documented. However, co-participation of the miRNA 5p/3p pairs in cancer cells has only recently begun to be recognized [14,35-38]. In this global analysis, we have identified 19 dysregulated 5p/3p pairs that are significantly co-expressed in colon cancer cells. Many of these miRNAs have previously been reported but without clear identification of which of the 5p/3p species is involved [18-21,39-42]. We further show that out of the 19 co-existing pairs, 17 pairs were either co-up- or co-down-regulated (Table 1), indicative of concerted selection of the 5’- and 3’-arm of the pre-miRNA precursors. Interestingly, two 5p/3p species showed reversed directions of expression in cancer cells.

There are increasing evidences to indicate selection of either or both the 5p or 3p alternative species under specific temporal, spatial and physiological and pathological conditions [3,4,7,9]. For arm selection, it has been proposed, based on thermodynamic consideration, that the strand with unstable base-pairing at the 5’-end is able to evade degradation [43,44]. Taking hints from reports that polymorphic indentation of a miRNA may lead to drastic shifts in arm selection to form novel miRNA isoforms (isomiRs) [45-47], the frequent co-expression of the 5p/3p pairs reported here in colon cancer cells may, to some extent, be driven by undetected polymorphic nucleotide changes in the miRNA genes in the cancer cells. In some cases, novel cancer-related extracellular signals may also lead to changes in strand selection in cancer cells [48], with reported changes in strand selection in different tissues and in different developmental stages when there are abundant spatial- and temporal-specific signals [3-5,7]. In different types of cancer, Dicer expression is dysregulated [49-52], making an impact on miRNA strand selection and maturation. Upon loading, Ago2 cleaves the passenger strand or repress translation of the targeted transcript [53,54]. Although Ago2 alone may be sufficient for strand selection for some miRNAs, it is demonstrated that for others, strand selection is also dependent on the availability of another RISC enzyme, the double-stranded RNA binding protein (dsRBP) [55]. Out of the eight human Ago proteins analyzed in colon cancer cells, at least two members have been found to be over-expressed in cancer tissues [56]. Likewise, subtle changes in the steady-state levels of dsRBP and other dsRBP-associating enzymes [55] would have effects on 5p/3p strand selection of concurrent expression in cancer cells. In summary, co-regulation of the 5p/3p miRNA species in cancer cells is probably multi-faceted, and may be subjected to subtle pathophysiological changes pre-miRNA processing enzymes in cancer cells.

In an analysis of six 5p/3p pairs and four putative targets (Figure 1), it is noted that miRNAs of the same family may target the same (e.g. let-7g-5p and let-7d-5p both targeting IGF1R), or different targets (e.g. let-7g and let-7d targeting IGF1R, KRAS and THBS1) of the metastasis process, and that a target transcript in this process may also be regulated by different miRNA species (e.g. KRAS is targeted by let-7d-3p, miR-18-3p and miR-30-3p) (Figure 1 and Tables 4 and 5). miRNA cross-regulation of targets of related biological function suggests a fail-proof mode of miRNA regulation in cancer cells to ensure that when any one of the regulatory miRNAs is disabled by mutations or by transcriptional or post-transcriptional suppression, other miRNA species in the regulatory circuit are still available to exert the affected biological function. Co-participation of the 5p/3p species adds further advantages to the fail-proof execution of miRNA regulation. Our proposition is consistent with numerous experimentally validated 5p/3p pairs that often suppress transcripts in related cancer pathways (e.g. see [14,37,38]).

The let-7 family miRNAs is a group of key regulators of differentiation processes in development; changes in let-7 expression could lead to dedifferentiation via EMT and tumor progression [57]. Let-7 down-regulation is associated with the development of a number of human cancers; let-7 is, hence, designated as a tumor suppressive miRNA. In this work, we present evidences to indicate targeting of IGF1R by let-7d-5p in the regulation of IGF1R in colon cancer cells. IGF1R, a transmembrane tyrosine kinase, is an integral component of the insulin-like growth factor system that regulates apoptosis, cell proliferation and transformation [58-61]. IGF1R is abundantly expressed in normal colorectal cells and in early-stage colon cancers but the expression is down-regulated in advanced-stage invasive colorectal cancers [62-65]. Besides let-7, a flurry of recent papers has reported no fewer than ten miRNAs down-regulate IGF1R in various types of cancers (see Additional file 6 for a list of references). IGF1R has become a crucial receptor protein in understanding tumor metastasis, and, hence, a therapeutic target. *Let-7d* regulation of KRAS has also previously been shown [66-68]. KRAS is a key signalling protein involved in apoptosis, proliferation and differentiation. In KRAS-transformed cells, the oncoprotein results in changes in cell adhesion and migration properties, and, thus, the metastatic potential of the cancer cells [69-71]. In this report, we show that it is let-7d-3p that is regulating KRAS. KRAS has also been experimental validated to be regulated by about a dozen other miRNAs (Additional file 6). Taken together, the observation of let-7d-5p/3p and multiple-miRNA targeting of IGF1R and KRAS is consistent with the safe-proof mechanism of miRNA regulation of crucial factors in cancer-related pathways to warranty continued advantages in the promotion and maintenance of the cancer phenotype.

## Conclusions

In this work, frequent co-expression and concerted regulation of miRNA-5p/3p pairs is demonstrated in colon cancer cells. Some 5p/3p species were found to target the same transcript and the same miRNA may cross-target different transcripts of proteins of the same biological process in a fail-proof scheme of miRNA regulation. Our data suggest the importance in further elucidation of possible clinical significance of co-existing miRNA-5p/3p pairs in cancers and other human diseases.

## Additional files

Additional file 1:
**Old and new nomenclatures and sequences of miRNAs described in this work.** According to miRBase Release 19, the old and new nomenclatures and sequences of miRNAs are listed. Additional file 2:
**Oligonucleotides used in stem-loop RT-PCR.** The sequences of the oligonucleotide primers used in the stem-loop RT-PCR validation of 5p/3p co-expression are shown. Additional file 3:
**Oligonucleotide primers for luciferase constructs and mutagenesis.** Two sub-tables are shown: A. Primers for luciferase constructs harboring let-7d-5p putative target sites in IFG1R. B. Oligonucleotides used for site-directed mutagenesis. Additional file 4:
**Number of predicted miRNA-targeted transcripts.** The number of target sites of the listed miRNA was calculated based on microRNA.org, DIANA-microT and miRDB database. Additional file 5:
**Sister strands of miRNAs and affected target transcripts of factors involved in the metastasis-associated processes in colon cancer cells (n = 19 pairs).** The affected target transcripts by both miRNA strands. The miRNA algorithms used in data derivation included microRNA.org (www.microrna.org); miRBase (www.miRBase.org), DIANA LAB - DNA Intelligent Analysis - TarBase Web Server and microT v4.0 Web Server; miRWalk (www.ma.uni-heidelberg.de/apps/zmf/mirwalk/); KEGG pathway database (www.genome.jp/kegg/pathway.html). Additional file 6:
**Experimentally-validated miRNAs that have been reported to regulate KRAS and IGF1R.** The references of the reported miRNAs targeting KRAS and IGF1R are listed.

## Abbreviations

EMT: Epithelial-mesenchymal transition
miRNA: microRNA
RISC: RNA-induced silencing complex
THBS1: Thrombospondin 1
TP53: Tumor suppressor protein 53
ZEB1/2: Zinc finger E-box binding homeobox 1 and 2

## References

[1] Yang JS, Lai EC (2011). Alternative miRNA biogenesis pathways and the interpretation of core miRNA pathway mutants. Mol Cell.

[2] Winter J, Jung S, Keller S, Gregory RI, Diederichs S (2009). Many roads to maturity: microRNA biogenesis pathways and their regulation. Nat Cell Biol.

[3] Ro S, Park C, Young D, Sanders KM, Yan W (2007). Tissue-dependent paired expression of miRNAs. Nucleic Acids Res.

[4] Jagadeeswaran G, Zheng Y, Sumathipala N, Jiang H, Arrese EL, Soulages JL, Zhang W, Sunkar R (2010). Deep sequencing of small RNA libraries reveals dynamic regulation of conserved and novel microRNAs and microRNA-stars during silkworm development. BMC Genomics.

[5] Griffiths-Jones S, Hui JH, Marco A, Ronshaugen M (2011). MicroRNA evolution by arm switching. EMBO Rep.

[6] Kuchenbauer F, Mah SM, Heuser M, McPherson A, Ruschmann J, Rouhi A, Berg T, Bullinger L, Argiropoulos B, Morin RD, Lai D, Starczynowski DT, Karsan A, Eaves CJ, Watahiki A, Wang Y, Aparicio SA, Ganser A, Krauter J, Döhner H, Döhner K, Marra MA, Camargo FD, Palmqvist L, Buske C, Humphries RK (2011). Comprehensive analysis of mammalian miRNA* species and their role in myeloid cells. Blood.

[7] Yang JS, Phillips MD, Betel D, Mu P, Ventura A, Siepel AC, Chen KC, Lai EC (2011). Widespread regulatory activity of vertebrate microRNA* species. RNA.

[8] Okamura K, Phillips MD, Tyler DM, Duan H, Chou YT, Lai EC (2008). The regulatory activity of microRNA* species has substantial influence on microRNA and 3’ UTR evolution. Nat Struct Mol Biol.

[9] Guo L, Lu Z (2010). The fate of miRNA* strand through evolutionary analysis: implication for degradation as merely carrier strand or potential regulatory molecule?. PLoS One.

[10] Jazdzewski K, Liyanarachchi S, Swierniak M, Pachucki J, Ringel MD, Jarzab B, de la Chapelle A (2009). Polymorphic mature microRNAs from passenger strand of pre-miR-146a contribute to thyroid cancer. Proc Natl Acad Sci U S A.

[11] Jiang L, Huang Q, Zhang S, Zhang Q, Chang J, Qiu X, Wang E (2010). Hsa-miR-125a-3p and hsa-miR-125a-5p are downregulated in non-small cell lung cancer and have inverse effects on invasion and migration of lung cancer cells. BMC Cancer.

[12] Almeida MI, Nicoloso MS, Zeng L, Ivan C, Spizzo R, Gafa R, Xiao L, Zhang X, Vannini I, Fanini F, Fabbri M, Lanza G, Reis RM, Zweidler-McKay PA, Calin GA (2012). Strand-specific miR-28-5p and miR-28-3p have distinct effects in colorectal cancer cells. Gastroenterology.

[13] Martin EC, Elliott S, Rhodes LV, Antoon JW, Fewell C, Zhu Y, Driver JL, Jodari-Karimi M, Taylor CW, Flemington EK, Beckman BS, Collins-Burow BM, Burow ME (2012). Preferential star strand biogenesis of pre-miR-24-2 targets PKC-alpha and suppresses cell survival in MCF-7 breast cancer cells. Mol Carcinog.

[14] Shan SW, Fang L, Shatseva T, Rutnam ZJ, Yang X, Du W, Lu WY, Xuan JW, Deng Z, Yang BB (2013). Mature miR-17-5p and passenger miR-17-3p induce hepatocellular carcinoma by targeting PTEN, GalNT7 and vimentin in different signal pathways. J Cell Sci.

[15] Koh W, Sheng CT, Tan B, Lee QY, Kuznetsov V, Kiang LS, Tanavde V (2010). Analysis of deep sequencing microRNA expression profile from human embryonic stem cells derived mesenchymal stem cells reveals possible role of let-7 microRNA family in downstream targeting of hepatic nuclear factor 4 alpha. BMC Genomics.

[16] Huang X, Gschweng E, Van Handel B, Cheng D, Mikkola HK, Witte ON (2011). Regulated expression of microRNAs-126/126* inhibits erythropoiesis from human embryonic stem cells. Blood.

[17] Parkin DM, Bray F, Ferlay J, Pisani P (2005). Global cancer statistics, 2002. CA Cancer J Clin.

[18] Slaby O, Svoboda M, Michalek J, Vyzula R (2009). MicroRNAs in colorectal cancer: translation of molecular biology into clinical application. Mol Cancer.

[19] de Krijger I, Mekenkamp LJ, Punt CJ, Nagtegaal ID (2011). MicroRNAs in colorectal cancer metastasis. J Pathol.

[20] Panarelli NC, Yantiss RK (2011). MicroRNA Expression in Selected Carcinomas of the Gastrointestinal Tract. Patholog Res Int.

[21] Wu WK, Law PT, Lee CW, Cho CH, Fan D, Wu K, Yu J, Sung JJ (2011). MicroRNA in colorectal cancer: from benchtop to bedside. Carcinogenesis.

[22] Huang CJ, Nguyen PN, Choo KB, Sugii S, Wee K, Cheong SK, Kamarul T (2014). Frequent Co-expression of miRNA-5p and -3p species and cross-targeting in induced pluripotent stem cells. Int J Med Sci.

[23] Choo KB, Hsu MC, Tsai YH, Lin WY, Huang CJ (2011). Nuclear factor kappa B and tumor necrosis factor-alpha modulation of transcription of the mouse testis- and pre-implantation development-specific Rnf33/Trim60 gene. FEBS J.

[24] Huang CJ, Chen HY, Lin WY, Choo KB (2014). Differential expression of speckled POZ protein, SPOP: putative regulation by miR-145. J Biosci.

[25] Wang M, Vogel I, Kalthoff H (2003). Correlation between metastatic potential and variants from colorectal tumor cell line HT-29. World J Gastroenterol.

[26] de Toledo M, Anguille C, Roger L, Roux P, Gadea G (2012). Cooperative anti-invasive effect of Cdc42/Rac1 activation and ROCK inhibition in SW620 colorectal cancer cells with elevated blebbing activity. PLoS One.

[27] Nicoloso MS, Spizzo R, Shimizu M, Rossi S, Calin GA (2009). MicroRNAs–the micro steering wheel of tumour metastases. Nat Rev Cancer.

[28] Cong N, Du P, Zhang A, Shen F, Su J, Pu P, Wang T, Zjang J, Kang C, Zhang Q (2013). Downregulated microRNA-200a promotes EMT and tumor growth through the wnt/beta-catenin pathway by targeting the E-cadherin repressors ZEB1/ZEB2 in gastric adenocarcinoma. Oncol Rep.

[29] Mongroo PS, Rustgi AK (2010). The role of the miR-200 family in epithelial-mesenchymal transition. Cancer Biol Ther.

[30] Biasiolo M, Sales G, Lionetti M, Agnelli L, Todoerti K, Bisognin A, Coppe A, Romualdi C, Neri A, Bortoluzzi S (2011). Impact of host genes and strand selection on miRNA and miRNA* expression. PLoS One.

[31] Olive V, Jiang I, He L (2010). mir-17-92, a cluster of miRNAs in the midst of the cancer network. Int J Biochem Cell Biol.

[32] Hafner M, Landthaler M, Burger L, Khorshid M, Hausser J, Berninger P, Rothballer A, Ascano M, Jungkamp AC, Munschauer M, Ulrich A, Wardle GS, Dewell S, Zavolan M, Tuschl T (2010). Transcriptome-wide identification of RNA-binding protein and microRNA target sites by PAR-CLIP. Cell.

[33] Yu ML, Wang JF, Wang GK, You XH, Zhao XX, Jing Q, Qin YW (2011). Vascular smooth muscle cell proliferation is Influenced by let-7d microRNA and its interaction with KRAS. Circ J.

[34] Jiao LR, Frampton AE, Jacob J, Pellegrino L, Krell J, Giamas G, Tsim N, Vlavianos P, Cohen P, Ahmad R, Keller A, Habib NA, Stebbing J, Castellano L (2012). MicroRNAs targeting oncogenes are down-regulated in pancreatic malignant transformation from benign tumors. PLoS One.

[35] Mah SM, Buske C, Humphries RK, Kuchenbauer F (2010). miRNA*: a passenger stranded in RNA-induced silencing complex?. Crit Rev Eukaryot Gene Expr.

[36] Lujambio A, Lowe SW (2012). The microcosmos of cancer. Nature.

[37] Zhang T, Luo Y, Wang T, Yang JY (2012). MicroRNA-297b-5p/3p target Mllt3/Af9 to suppress lymphoma cell proliferation, migration and invasion in vitro and tumor growth in nude mice. Leuk Lymphoma.

[38] Zhang Y, Yang P, Sun T, Li D, Xu X, Rui Y, Li C, Chong M, Ibrahim T, Mercatali L, Amadori D, Lu X, Xie D, Li QJ, Wang XF (2013). miR-126 and miR-126* repress recruitment of mesenchymal stem cells and inflammatory monocytes to inhibit breast cancer metastasis. Nat Cell Biol.

[39] Bojmar L, Karlsson E, Ellegård S, Olsson H, Björnsson B, Hallböök O, Larsson M, Stål O, Sandström P (2013). The role of microRNA-200 in progression of human colorectal and breast cancer. PLoS One.

[40] Chen WS, Chen TW, Yang TH, Hu LY, Pan HW, Leung CM, Li SC, Ho MR, Shu CW, Liu PF, Yu SY, Tu YT, Lin WC, Wu TT, Tsai KW (2013). Co-modulated behavior and effects of differentially expressed miRNA in colorectal cancer. BMC Genomics.

[41] Drusco A, Nuovo GJ, Zanesi N, Di Leva G, Pichiorri F, Volinia S, Fernandez C, Antenucci A, Costinean S, Bottoni A, Rosito IA, Liu CG, Burch A, Acunzo M, Pekarsky Y, Alder H, Ciardi A, Croce CM (2014). MicroRNA profiles discriminate among colon cancer metastasis. PLoS One.

[42] Zanutto S, Pizzamiglio S, Ghilotti M, Bertan C, Ravagnani F, Perrone F, Leo E, Pilotti S, Verderio P, Gariboldi M, Pierotti MA (2014). Circulating miR-378 in plasma: a reliable, haemolysis-independent biomarker for colorectal cancer. Br J Cancer.

[43] Khvorova A, Reynolds A, Jayasena SD (2003). Functional siRNAs and miRNAs exhibit strand bias. Cell.

[44] Schwarz DS, Hutvagner G, Du T, Xu Z, Aronin N, Zamore PD (2003). Asymmetry in the assembly of the RNAi enzyme complex. Cell.

[45] Ohanian M, Humphreys DT, Anderson E, Preiss T, Fatkin D (2013). A heterozygous variant in the human cardiac miR-133 gene, MIR133A2, alters miRNA duplex processing and strand abundance. BMC Genet.

[46] Chan YT, Lin YC, Lin RJ, Kuo HH, Thang WC, Chiu KP, Yu AL (2013). Concordant and discordant regulation of target genes by miR-31 and its isoforms. PLoS One.

[47] Llorens F, Banez-Coronel M, Pantano L, Del Rio JA, Ferrer I, Estivill X, Marti E (2013). A highly expressed miR-101 isomiR is a functional silencing small RNA. BMC Genomics.

[48] Marco A, Macpherson JI, Ronshaugen M, Griffiths-Jones S (2012). MicroRNAs from the same precursor have different targeting properties. Silence.

[49] Chiosea S, Jelezcova E, Chandran U, Acquafondata M, McHale T, Sobol RW, Dhir R (2006). Up-regulation of dicer, a component of the MicroRNA machinery, in prostate adenocarcinoma. Am J Pathol.

[50] Wu D, Tao J, Xu B, Li P, Lu Q, Zhang W (2012). Downregulation of Dicer, a component of the microRNA machinery, in bladder cancer. Mol Med Rep.

[51] Merritt WM, Lin YG, Han LY, Kamat AA, Spannuth WA, Schmandt R, Urbauer D, Pennacchio LA, Cheng JF, Nick AM, Deavers MT, Mourad-Zeidan A, Wang H, Mueller P, Lenburg ME, Gray JW, Mok S, Birrer MJ, Lopez-Berestein G, Coleman RL, Bar-Eli M, Sood AK (2008). Dicer, Drosha, and outcomes in patients with ovarian cancer. N Engl J Med.

[52] Grelier G, Voirin N, Ay AS, Cox DG, Chabaud S, Treilleux I, Leon-Goddard S, Rimokh R, Mikaelian I, Venoux C, Puisieux A, Lasset C, Moyret-Lalle C (2009). Prognostic value of Dicer expression in human breast cancers and association with the mesenchymal phenotype. Br J Cancer.

[53] Wang B, Li S, Qi HH, Chowdhury D, Shi Y, Novina CD (2009). Distinct passenger strand and mRNA cleavage activities of human Argonaute proteins. Nat Struct Mol Biol.

[54] Ghildiyal M, Xu J, Seitz H, Weng Z, Zamore PD (2010). Sorting of Drosophila small silencing RNAs partitions microRNA* strands into the RNA interference pathway. RNA.

[55] Noland CL, Doudna JA (2013). Multiple sensors ensure guide strand selection in human RNAi pathways. RNA.

[56] Li L, Yu C, Gao H, Li Y (2010). Argonaute proteins: potential biomarkers for human colon cancer. BMC Cancer.

[57] Peter ME (2009). Let-7 and miR-200 microRNAs: guardians against pluripotency and cancer progression. Cell Cycle.

[58] Bustin SA, Jenkins PJ (2001). The growth hormone-insulin-like growth factor-I axis and colorectal cancer. Trends Mol Med.

[59] Hassan AB, Macaulay VM (2002). The insulin-like growth factor system as a therapeutic target in colorectal cancer. Ann Oncol.

[60] Moschos SJ, Mantzoros CS (2002). The role of the IGF system in cancer: from basic to clinical studies and clinical applications. Oncology.

[61] Reinmuth N, Liu W, Fan F, Jung YD, Ahmad SA, Stoeltzing O, Bucana CD, Radinsky R, Ellis LM (2002). Blockade of insulin-like growth factor I receptor function inhibits growth and angiogenesis of colon cancer. Clin Cancer Res.

[62] Hakam A, Yeatman TJ, Lu L, Mora L, Marcet G, Nicosia SV, Karl RC, Coppola D (1999). Expression of insulin-like growth factor-1 receptor in human colorectal cancer. Hum Pathol.

[63] Peters G, Gongoll S, Langner C, Mengel M, Piso P, Klempnauer J, Ruschoff J, Kreipe H, von Wasielewski R (2003). IGF-1R, IGF-1 and IGF-2 expression as potential prognostic and predictive markers in colorectal-cancer. Virchows Arch.

[64] Freier S, Weiss O, Eran M, Flyvbjerg A, Dahan R, Nephesh I, Safra T, Shiloni E, Raz I (1999). Expression of the insulin-like growth factors and their receptors in adenocarcinoma of the colon. Gut.

[65] Allison AS, McIntyre MA, McArdle C, Habib FK (2007). The insulin-like growth factor type 1 receptor and colorectal neoplasia: insights into invasion. Hum Pathol.

[66] Johnson SM, Grosshans H, Shingara J, Byrom M, Jarvis R, Cheng A, Labourier E, Reinert KL, Brown D, Slack FJ (2005). RAS is regulated by the let-7 microRNA family. Cell.

[67] Smits KM, Paranjape T, Nallur S, Wouters KA, Weijenberg MP, Schouten LJ, van den Brandt PA, Bosman FT, Weidhaas JB, van Engeland M (2011). A let-7 microRNA SNP in the KRAS 3’UTR is prognostic in early-stage colorectal cancer. Clin Cancer Res.

[68] Li ZH, Pan XM, Han BW, Guo XM, Zhang Z, Jia J, Gao LB (2013). A let-7 binding site polymorphism rs712 in the KRAS 3’ UTR is associated with an increased risk of gastric cancer. Tumour Biol.

[69] Jancik S, Drabek J, Radzioch D, Hajduch M (2010). Clinical relevance of KRAS in human cancers. J Biomed Biotechnol.

[70] Esser D, Bauer B, Wolthuis RM, Wittinghofer A, Cool RH, Bayer P (1998). Structure determination of the Ras-binding domain of the Ral-specific guanine nucleotide exchange factor Rlf. Biochemistry.

[71] Zuber J, Tchernitsa OI, Hinzmann B, Schmitz AC, Grips M, Hellriegel M, Sers C, Rosenthal A, Schafer R (2000). A genome-wide survey of RAS transformation targets. Nat Genet.

