# Supplementary material for: MicroRNA-5p and -3p co-expression and cross-targeting in colon cancer cells
Source: J Biomed Sci. 2014 Oct 5;21(1):95. doi: 10.1186/s12929-014-0095-x (PMC4195866; doi:10.1186/s12929-014-0095-x)
Supplement: Additional file 1: — Old and new nomenclatures and sequences of miRNAs described in this work. According to miRBase Release 19, the old and new nomenclatures and sequences of miRNAs are listed. [file 12929_2014_95_MOESM1_ESM.docx]

Additional file 1. Old and new nomenclatures and sequences of miRNAs described in this work

| miRNA^1^ | Other names^1^ | Sequence (5’ to 3’) |
| --- | --- | --- |
| hsa-let-7d-5p  hsa-let-7d-3p | hsa-let-7d  hsa-let-7d* | AGAGGUAGUAGGUUGCAUAGUU  CUAUACGACCUGCUGCCUUUCU |
| hsa-let-7g-5p  hsa-let-7g-3p | hsa-let-7g  hsa-let-7g* | UGAGGUAGUAGUUUGUACAGUU  CUGUACAGGCCACUGCCUUGC |
| hsa-let-7i-5p  hsa-let-7i-3p | hsa-let-7i  hsa-let-7i* | UGAGGUAGUAGUUUGUGCUGUU  CUGCGCAAGCUACUGCCUUGCU |
| hsa-miR-7-5p  hsa-miR-7-1-3p | hsa-miR-7  hsa-miR-7-1* | UGGAAGACUAGUGAUUUUGUUGU  CAACAAAUCACAGUCUGCCAUA |
| hsa-miR-17-5p  hsa-miR-17-3p | hsa-miR-17  hsa-miR-17* | CAAAGUGCUUACAGUGCAGGUAG  ACUGCAGUGAAGGCACUUGUAG |
| hsa-miR-18a-5p  hsa-miR-18a-3p | hsa-miR-18; hsa-miR-18a  hsa-miR-18a* | UAAGGUGCAUCUAGUGCAGAUAG  ACUGCCCUAAGUGCUCCUUCUGG |
| hsa-miR-20a-5p  hsa-miR-20a-3p | hsa-miR-20; hsa-miR-20a  hsa-miR-20a* | UAAAGUGCUUAUAGUGCAGGUAG  ACUGCAUUAUGAGCACUUAAAG |
| hsa-miR-21-5p  hsa-miR-21-3p | hsa-miR-21  hsa-miR-21* | UAGCUUAUCAGACUGAUGUUGA  CAACACCAGUCGAUGGGCUGU |
| hsa-miR-22-5p  hsa-miR-22-3p | hsa-miR-22*  hsa-miR-22 | AGUUCUUCAGUGGCAAGCUUUA  AAGCUGCCAGUUGAAGAACUGU |
| hsa-miR-27a-5p  hsa-miR-27a-3p | hsa-miR-27a*  hsa-miR-27a | AGGGCUUAGCUGCUUGUGAGCA  UUCACAGUGGCUAAGUUCCGC |
| hsa-miR-29b-1-5p  hsa-miR-29b-3p | hsa-miR-29b-1*  hsa-miR-29b | GCUGGUUUCAUAUGGUGGUUUAGA  UAGCACCAUUUGAAAUCAGUGUU |
| hsa-miR-31-5p  hsa-miR-31-3p | hsa-miR-31  hsa-miR-31* | AGGCAAGAUGCUGGCAUAGCU  UGCUAUGCCAACAUAUUGCCAU |
| hsa-miR-141-5p  hsa-miR-141-3p | hsa-miR-141*  hsa-miR-141 | CAUCUUCCAGUACAGUGUUGGA  UAACACUGUCUGGUAAAGAUGG |
| hsa-miR-151a-5p  hsa-miR-151a-3p | hsa-miR-151-5p  hsa-miR-151; hsa-miR-151-3p | UCGAGGAGCUCACAGUCUAGU |
|  |  | CUAGACUGAAGCUCCUUGAGG |
| hsa-miR-199a-5p  hsa-miR-199a-3p | hsa-miR-199a  hsa-miR-199a* | CCCAGUGUUCAGACUACCUGUUC  ACAGUAGUCUGCACAUUGGUUA |
| hsa-miR-200a-5p  hsa-miR-200a-3p | hsa-miR-200a*  hsa-miR-200a | CAUCUUACCGGACAGUGCUGGA  UAACACUGUCUGGUAACGAUGU |
| hsa-miR-200b-5p  hsa-miR-200b-3p | hsa-miR-200b*  hsa-miR-200b | CAUCUUACUGGGCAGCAUUGGA  UAAUACUGCCUGGUAAUGAUGA |
| hsa-miR-378a-5p  hsa-miR-378a-3p | hsa-miR-378; hsa-miR-378*  hsa-miR-378 | CUCCUGACUCCAGGUCCUGUGU  ACUGGACUUGGAGUCAGAAGG |
| hsa-miR-574-5p  hsa-miR-574-3p | none  hsa-miR-574 | UGAGUGUGUGUGUGUGAGUGUGU  CACGCUCAUGCACACACCCACA |

^1^Based on miRBase Release 19.
