# Supplementary material for: MicroRNA-5p and -3p co-expression and cross-targeting in colon cancer cells
Source: J Biomed Sci. 2014 Oct 5;21(1):95. doi: 10.1186/s12929-014-0095-x (PMC4195866; doi:10.1186/s12929-014-0095-x)
Supplement: Additional file 2: — Oligonucleotides used in stem-loop RT-PCR. The sequences of the oligonucleotide primers used in the stem-loop RT-PCR validation of 5p/3p co-expression are shown. [file 12929_2014_95_MOESM2_ESM.docx]

Additional file 2. Oligonucleotides used in stem-loop RT-PCR

__________________________________________________________________________________________________________________________

miRNA Stem-loop RT primers (5’-3’) ^a^ Forward primer (5’-3’) ^a^

__________________________________________________________________________________________________________________________

miR-21-5p GTCGTATCCAGTGCAGGGTCCGAGGTATTCGCACTGGATACGAC**TCAACATC** CGCGGCC**TAGCTTATCAGACTGAT**

miR-21-3p GTCGTATCCAGTGCAGGGTCCGAGGTATTCGCACTGGATACGAC**ACAGCCCA** GGC**CAACACCAGTCGATGGG**

miR-17-5p GTCGTATCCAGTGCAGGGTCCGAGGTATTCGCACTGGAT ACGAC**CTACCTGC** CGCG**CAA AGTGCTTACAGTGC**

miR-17-3p GTCGTATCCAGTGCAGGGTCCGAGGTATTCGCACTGGAT ACGAC**CTACAAGT** CGC**ACTGCAGTGAAGGCACT**

miR-141-5p GTCGTATCCAGTGCAGGGTCCGAGGTATTCGCACTGGAT ACGAC**TCCAACAC** CGCGC**CATCTTCCAGTACAGTG**

miR-141-3p GTCGTATCCAGTGCAGGGTCCGAGGTATTCGCACTGGAT ACGAC**CCATCTTT** GCCGCGC**TAACACTGTCTGGTA AA**

**__________________________________________________________________________________________________________________________**

^a^Sequences in bold and underscored are complementary to 3’ end of mature miRNA; the remaining sequence is the stem-loop sequence.
