# Supplementary material for: MicroRNA-5p and -3p co-expression and cross-targeting in colon cancer cells
Source: J Biomed Sci. 2014 Oct 5;21(1):95. doi: 10.1186/s12929-014-0095-x (PMC4195866; doi:10.1186/s12929-014-0095-x)
Supplement: Additional file 3: — Oligonucleotide primers for luciferase constructs and mutagenesis. Two sub-tables are shown: A. Primers for luciferase constructs harboring let-7d-5p putative target sites in IFG1R. B. Oligonucleotides used for site-directed mutagenesis. [file 12929_2014_95_MOESM3_ESM.docx]

Addition file 3. Oligonucleotide primers for luciferase constructs and mutagenesis.

**A. Primers for luciferase constructs harboring let-7d-5p putative target sites in IFG1R**

_________________________________________________________________________________________________________________

Clone Primer Sequences^a^ PCR 3’-UTR location^b^

Products

_________________________________________________________________________________________________________________

IGF1R-1 IGF1R-3’UTR-7dF1 5’- CATCTAGAATCTGTGCAAACAGTAACGTG -3’ 176 bp nt 4,169 – 4,344

IGF1R-3’UTR-7dR1 5’- GGTCTAGAGCATATTGAAAAAAGGAACATCC -3’

IGF1R-2 IGF1R-3’UTR-7dF2 5’- TTTCTAGATGTGTGGGGTGTGTGTGTGTGA -3’ 233 bp nt 6,698 – 6,930

IGF1R-3’UTR-7dR2 5’- TTTCTAGACCCCGGAATACAGCCTGGC -3’

IGF1R-3 IGF1R-3’UTR-7dF3 5’- CCTCTAGATTCATTTGGATGTTTGGCGTTGC -3’ 201 bp nt 10,697 – 10,897

IGF1R-3’UTR-7dR3 5’- TTTCTAGATGCATTATTTGCGGTGCATCCAT -3’

__________________________________________________________________________________________________________________

a. The XbaI restriction site used in the cloning is underlined. b. Based on *IGF1R* mRNA sequence: accession no. NM_000875

**B. Oligonucleotides used for site-directed mutagenesis**

_________________________________________________________________________________

Oligonucleotide Sequence^a^

_________________________________________________________________________________

IGF1R-1-mutF 5’- CACAAGCCTCCTG**cgCtgC**AGTGGATCTTCAG -3’

IGF1R-1-mutR 5’- CTGAAGATCCACT**GcaGcg**CAGGAGGCTTGTG -3’

IGF1R-2-mutF 5’- CCCAAACATTTAT**ggAatTC**caTCTTATTTTTTATATGTG -3’

IGF1R-2-mutR 5’- CACATATAAAAAATAAGAtg**GAatTcc**ATAAATGTTTGGG -3’

IGF1R-3-mutF 5’-GCCAGAGTTTGT**ggAatTCc**GGGTATCCCTTTGTC-3’

IGF1R-3-mutR 5’-GACAAAGGGATACCCg**GAatTcc**ACAAACTCTGGC-3

__________________________________________________________________________________

^a^The seed sequences are underlined. Mutated nucleotides are shown in bold and in lowercase letters.
