# Supplementary material for: MicroRNA-5p and -3p co-expression and cross-targeting in colon cancer cells
Source: J Biomed Sci. 2014 Oct 5;21(1):95. doi: 10.1186/s12929-014-0095-x (PMC4195866; doi:10.1186/s12929-014-0095-x)
Supplement: Additional file 4: — Number of predicted miRNA-targeted transcripts. The number of target sites of the listed miRNA was calculated based on microRNA.org, DIANA-microT and miRDB database. [file 12929_2014_95_MOESM4_ESM.docx]

Additional file 4. Number of predicted miRNA-targeted transcripts

_________________________________________________________________________________

miRNA microRNA.org DIANA-microT miRDB Mean (%)

(previous name)

_________________________________________________________________________________

1. ***let-7* family**

let-7g-5p (let-7g) 5544 (43.5%) 4909 (47.4%) 196 (25.7%) 44.6

let-7g-3p (let-7g*) 7204 (56.5%) 5453 (52.6%) 566 (74.3%) 55.4

let-7d-5p (let-7d) 5418 (81.8%) 4543 (79.8%) 208 (83.9%) 80.9

let-7d-3p (let-7d*) 1208 (18.2%) 1150 (20.2%) 40 (16.1%) 19.1

let-7i-5p (let-7i) 5577 (75.5%) 4828 (89.7%) 202 (90.6%) 81.6

let-7i-3p (let-7i*) 1809 (24.5%) 555 (10.3%) 21 (9.4%) 18.4

1. ***mir-8/200* family**

miR-200a-5p (miR-200a*) 5770 (43.0%) 5654 (53.5%) 727 (67.1%) 48.5

miR-200a-3p (miR-200a) 7645 (57.0%) 4910 (46.5%) 356 (32.9%) 51.5

miR-200b-5p (miR-200b*) 5849 (44.9%) 5804 (54.8%) 844 (71.3%) 50.4

miR-200b-3p (miR-200b) 7176 (55.1%) 4790 (45.2%) 340 (28.7%) 49.6

miR-141-5p (miR-141*) 7514 (49.3%) 5620 (45.9%) 710 (64.5%) 48.5

miR-141-3p (miR-141) 7721 (50.7%) 6620 (54.1%) 390 (35.5%) 51.5

1. ***mir-17* family**

miR-17-5p (miR-17) 9121 (47.9%) 7382 (49.9%) 950 (72.9%) 49.6

miR-17-3p (miR-17*) 9938 (52.1%) 7420 (50.1%) 353 (27.1%) 50.4

miR-18a-5p (miR-18a) 7381 (46.8%) 4482 (49.2%) 218 (45.8%) 47.6

miR-18a-3p (miR-18a*) 8387 (53.2%) 4631 (50.8%) 258 (54.2%) 52.4

miR-20a-5p (miR-20a) 9156 (54.7%) 7205 (55.6%) 887 (62.3%) 55.4

miR-20a-3p (miR-20a*) 7593 (45.3%) 5752 (44.4%) 537 (37.7%) 46.6

_________________________________________________________________________________
