# Supplementary material for: MicroRNA-5p and -3p co-expression and cross-targeting in colon cancer cells
Source: J Biomed Sci. 2014 Oct 5;21(1):95. doi: 10.1186/s12929-014-0095-x (PMC4195866; doi:10.1186/s12929-014-0095-x)
Supplement: Additional file 5: — Sister strands of miRNAs and affected target transcripts of factors involved in the metastasis-associated processes in colon cancer cells (n = 19 pairs). The affected target transcripts by both miRNA strands. The miRNA algorithms used in data derivation included microRNA.org (www.microrna.org); miRBase (www.miRBase.org), DIANA LAB - DNA Intelligent Analysis - TarBase Web Server and microT v4.0 Web Server; miRWalk (www.ma.uni-heidelberg.de/apps/zmf/mirwalk/); KEGG pathway database (www.genome.jp/kegg/pathway.html). [file 12929_2014_95_MOESM5_ESM.docx]

**­­­**Additional file 5. Sister strands of miRNAs and affected target transcripts of factors involved in the metastasis-associated processes in colon cancer cells (n = 19 pairs)

| No. | miRNA  (previous name) | Expression status | Log_2_(Fold change)  (mean+SD) | Affected processes: Selected target mRNAs |
| --- | --- | --- | --- | --- |
| 3 | hsa-miR-20a-5p  (hsa-miR-20p) | Up-regulated | 7.19±0.78** | Angiogenesis: CCND1, THBS1  Apoptosis: E2F1  Cell cycle: CCND1, E2F1, THBS1  p53 signalling pathway: CCND1, THBS1  VEGF-R signalling pathway: BMPR2  Wnt signalling pathway: CCND1 |
|  | hsa-miR-20a-3p  (hsa-miR-20a*) | Up-regulated | 1.58±0.98 | Angiogenesis: PPARA  Apoptosis: KRAS  Cancer: KRAS, MYB, PCNA  Cell cycle: PCNA  DNA repair: PCNA  Drug target: PTPRR  GABAergic synapse: SLC38A2  G-protein coupled receptor signalling pathway: LPAR1  MAPK signalling pathway: KRAS  Protein digestion and absorption: SLC38A2  VEGF signalling pathway: KRAS |
| 4 | hsa-miR-17-5p  (hsa-miR-17) | Up-regulated | 6.68±0.69** | Angiogenesis: CCND1, THBS1  Apoptosis: E2F1,  Cancer: E2F1, RBL2, THBS1  Cell cycle: CCND1, E2F1, RBL2, THBS1  MAPK signalling pathway: MAP3K2  Oxidative stress: MAP3K2  p53 signalling pathway: CCND1, THBS1  VEGF-R signalling pathway: BMPR2  Wnt signalling pathway: CCND1 |
|  | hsa-miR-17-3p  (hsa-miR-17*) | Up-regulated | 4.12±0.59** | Angiogenesis: CD44  Cancer: CD44, MYB  Inflammation: CD44, IL6ST  Lysine degradation: MLL3  Proteolysis: IFT52 |
| 5 | hsa-miR-7-1-5p  (hsa-miR-7) | Up-regulated | 6.44±1.14* | ABC transporters: ABCC1  Angiogenesis: CCND1, IGF1R, MYC, TIMP2  Apoptosis: EFGR, IGF1R, MYC  Cell cycle: CCND1  Cell growth: SLC3A2  Cell migration: EGFR, IRS1  DNA repair: EFGR  Inflammation: SLC7A5  MAPK signalling pathway: EFGR  Protein digestion and absorption: SLC3A2  Protein folding: CCT4  p53 signalling pathway: CCND1  Response to drug: ABCC1  Wnt signalling pathway: CCND1 |
|  | hsa-miR-7-1-3p  (hsa-miR-7-1*) | Up-regulated | 3.83±0.66** | Angiogenesis: IGF1R  Apoptosis: IGF1R  Cancer: IGF1R, JUN  Inflammation: JUN  MAPK signalling pathway: JUN  Wnt signalling pathway: JUN |
| 6 | hsa-miR-200a-5p  (hsa-miR-200a*) | Up-regulated | 5.17±0.65** | Apoptosis: BNIP1, FGF4, TIAM2  Cancer progression, invasion, metastasis: TIAM2  Chromatin modification: PRDM6  EMT: ZEB2  MAPK signalling pathway: FGF4  Wnt signalling pathway: FZD1, PPAP2B, ZEB2 |
|  | hsa-miR-200a-3p  (hsa-miR-200a) | Up-regulated | 8.10±1.58** | Angiogenesis: ITGB3  Cell migration: IRS2  Cell adhesion molecules(CAMs): SELE  EMT: ZEB1, ZEB2  Inflammation: SELE  Transcriptional misregulation in cancer: ZEB1  VEGF signalling pathway: ITGB3  Wnt signalling pathway: ZEB2 |
| 7 | hsa-miR-18a-5p  (hsa-miR-18a) | Up-regulated | 3.94±0.91** | Angiogenesis: HIF1A  Apoptosis: ATM  Cancer: SMAD2  Cell cycle: ATM, CCND2, CDK2, SMAD2  Cell migration: HIF1A  Cytokine-cytokine receptor interaction: TNFSF11  Focal adhesion: CCND2,CDC42, IGF1, MYLK, THBS1  MAPK signalling pathway: CACNB3, CDC42, DUSP16, MAP3K1, MEF2C, TAOK3, TNFSF11  p53 signalling pathway: ATM, CCND2, CDK2, IGF1, THBS1  TNF-mediated signalling pathway: TNFSF11  VEGF signalling pathway: CDC42, HIF1A  Wnt signalling pathway: CCND2, SMAD2 |
|  | hsa-miR-18a-3p  (hsa-miR-18a*) | Up-regulated | 3.18±0.77** | Apoptosis: CASP7, KRAS  Cancer: KRAS  Drug target: CASP7  MAPK signalling pathway: KRAS  VEGF signalling pathway: KRAS |
| 8 | hsa-miR-141-5p  (hsa-miR-141*) | Up-regulated | 3.85±0.76** | Apoptosis: ATM, NME1, ZAK  Cancer: ATM, NME1  Cell adhesion molecules(CAMs): CLDN1  Cell cycle: ATM  Inflammation: IL6R  Leukocyte transendothelial migration: CLDN1  Lysine degradation: MLL3  p53 signalling pathway: ATM |
|  | hsa-miR-141-3p  (hsa-miR-141) | Up-regulated | 4.50±1.08** | Angiogenesis: CCND1, ETS1, MAPK14, MET, MYC, PIK3CA, TP53  Apoptosis: MAP3K10, MYC, PPIA, TP53  Cancer: CCND1, CDC25C, JUN, MYB, MYC, PIK3CA, PPIA, TP53  Cell cycle: CCND1, TP53  Drug target: CDC25C, PPIA  EMT: ZEB1  Focal adhesion: MET  Inflammation: JUN, MAPK14, PPIA, TP53 MAPK signalling pathway: JUN, MAPK14, MAP3K10, TP53  p53 signalling pathway: CCND1, TP53  Transcriptional misregulation in cancer: ZEB1  Wnt signalling pathway: CCND1, JUN, TP53 |
| 9 | hsa-let-7g-5p  (hsa-let-7g) | Up-regulated | 2.97±0.49** | Angiogenesis: IGF1R, MYC, TNFSF10  Apoptosis: BCL2L1, IGF1R, MDM4, MYC, TNFSF10  Cancer: BCL2L1, COL1A1, HMGA1, IGF1R, MDM4, MYC  Cell cycle: MDM4  Inflammation: TNFSF10  p53 signalling pathway: MDM4 |
|  | hsa-let-7g-3p  (hsa-let-7g*) | Up-regulated | 2.12±0.13* | Angiogenesis: ITGA1  Apoptosis: EI24, ZAK  DNA integration/DNA mediated transformation: RLF  EMT: ZEB1  MAPK signalling pathway: NLK  Pyrimidine metabolism/ metabolic pathways: CAD  Transcriptional misregulation in cancer: ZEB1  Wnt signalling pathway: NLK |
| 10 | hsa-miR-27a-5p  (hsa-miR-27a*) | Up-regulated | 2.94±1.21** | ABC transporters: ABCA1  Angiogenesis: PDGFA  Apoptosis: ATM, GSPT1  Cancer: ATM, GSPT1, PDGFA  Cell cycle: ATM, PRMT5  Fat digestion and absorption: ABCA1  G-protein coupled receptor signalling pathway: ABCA1  Histone methylation: PRMT5  p53 signalling pathway: ATM  RNA transport: PRMT5 |
|  | hsa-miR-27a-3p  (hsa-miR-27a) | Up-regulated | 4.78±0.62** | Angiogenesis: CCND1, CTSD, EPHA2, IGF1R, MET, TP53  Apoptosis: ATM, FOXO1, IGF1R, TP53  Cancer: ATM, EPHA2, FOXO1, IGF1R, KPNA2, PCNA, TP53  Cell cycle: ATM, CCND1, PCNA, TP53  DNA repair: PCNA  Focal adhesion: MET  Inflammation: TP53  MAPK signalling pathway: TP53  p53 signalling pathway: ATM, CCND1, TP53  Wnt signalling pathway: CCND1, TP53 |
| 13 | hsa-miR-151a-5p  (hsa-miR-151-5p) | Up-regulated | 2.65±0.67* | Drug metabolism – cytochrome P450/ metabolic pathways: CYP2E1 |
|  | hsa-miR-151a-3p  (hsa-miR-151-3p) | Up-regulated | 5.39±1.20** | Angiogenesis: EFNB2, THBS1  Cancer: THBS1  Cell cycle: THBS1  EMT: ZEB1  Transcriptional misregulation in cancer: ZEB1 |
| 15 | hsa-let-7d-5p  (hsa-let-7d) | Up-regulated | 2.29±1.01 | Angiogenesis: IGF1R  Apoptosis: IGF1R  Cancer: IGF1R |
|  | hsa-let-7d-3p  (hsa-let-7d*) | Down-regulated | -2.78±0.79** | Apoptosis: KRAS  Cancer: KRAS |
| 16 | hsa-miR-200b-5p  (hsa-miR-200b*) | Down-regulated | -1.67±0.39 | Chromatin modification: PRDM6  EMT: ZEB2  Small GTPase mediated signal transduction/ GTP catabolic process: RAB1A  Wnt signalling pathway: ZEB2 |
|  | hsa-miR-200b-3p  (hsa-miR-200b) | Up-regulated | 3.15±0.25** | Cancer: MLH1  EMT: ZEB1  Mismatch repair: MLH1  Transcriptional misregulation in cancer: ZEB1 |

**miRNA algorithms used in data derivation:** microRNA.org ([www.microrna.org](http://www.microrna.org)); miRBase ([www.miRBase.org](http://www.miRBase.org)), DIANA LAB - DNA Intelligent Analysis - TarBase Web Server and microT v4.0 Web Server; miRWalk ([www.ma.uni-heidelberg.de/apps/zmf/mirwalk/](http://www.ma.uni-heidelberg.de/apps/zmf/mirwalk/)); KEGG pathway database ([www.genome.jp/kegg/pathway.html](http://www.genome.jp/kegg/pathway.html))
