# Supplementary material for: MicroRNA-5p and -3p co-expression and cross-targeting in colon cancer cells
Source: J Biomed Sci. 2014 Oct 5;21(1):95. doi: 10.1186/s12929-014-0095-x (PMC4195866; doi:10.1186/s12929-014-0095-x)
Supplement: Additional file 6: — Experimentally-validated miRNAs that have been reported to regulate KRAS and IGF1R. The references of the reported miRNAs targeting KRAS and IGF1R are listed. [file 12929_2014_95_MOESM6_ESM.docx]

Additional file 6. Experimentally validated miRNAs that regulate KRAS and IGF1R in the literature

_________________________________________________________________________________________________________________________________

**miRNA References**

_________________________________________________________________________________________________________________________________

1. **KRAS**

let-7c Levy R, Biran A, Poirier F, Raz A, Kloog Y. 2011. [Galectin-3 mediates cross-talk between K-Ras and Let-7c tumor suppressor microRNA.](http://www.ncbi.nlm.nih.gov/pubmed/22102901) PLoS One. 6:e27490.

miR-21 Ren J, Zhu D, Liu M, Sun Y, Tian L. 2010. [Downregulation of miR-21 modulates Ras expression to promote apoptosis and suppress invasion of Laryngeal squamous cell carcinoma.](http://www.ncbi.nlm.nih.gov/pubmed/20801640) Eur J Cancer. 46:3409-3416.

miR-30c Tanic M, Yanowsky K, Rodriguez-Antona C, Andre´s R, Ma´rquez-Rodas I, et al. 2012. Deregulated miRNAs in hereditary breast cancer revealed a role for miR-30c in regulating KRAS oncogene. PLoS One. 7: e38847.

miR-96 [Yu S](http://www.ncbi.nlm.nih.gov/pubmed?term=Yu%20S%5BAuthor%5D&cauthor=true&cauthor_uid=20610624), [Lu Z](http://www.ncbi.nlm.nih.gov/pubmed?term=Lu%20Z%5BAuthor%5D&cauthor=true&cauthor_uid=20610624), [Liu C](http://www.ncbi.nlm.nih.gov/pubmed?term=Liu%20C%5BAuthor%5D&cauthor=true&cauthor_uid=20610624), [Meng Y](http://www.ncbi.nlm.nih.gov/pubmed?term=Meng%20Y%5BAuthor%5D&cauthor=true&cauthor_uid=20610624), [Ma Y](http://www.ncbi.nlm.nih.gov/pubmed?term=Ma%20Y%5BAuthor%5D&cauthor=true&cauthor_uid=20610624), [Zhao W](http://www.ncbi.nlm.nih.gov/pubmed?term=Zhao%20W%5BAuthor%5D&cauthor=true&cauthor_uid=20610624), [Liu J](http://www.ncbi.nlm.nih.gov/pubmed?term=Liu%20J%5BAuthor%5D&cauthor=true&cauthor_uid=20610624), [Yu J](http://www.ncbi.nlm.nih.gov/pubmed?term=Yu%20J%5BAuthor%5D&cauthor=true&cauthor_uid=20610624), [Chen J](http://www.ncbi.nlm.nih.gov/pubmed?term=Chen%20J%5BAuthor%5D&cauthor=true&cauthor_uid=20610624). 2010. miRNA-96 suppresses KRAS and functions as a tumor suppressor gene in pancreatic cancer. [Cancer Res.](http://www.ncbi.nlm.nih.gov/pubmed/20610624) 70:6015-6025.

miR-143 Xu B, Niu X, Zhang X, Tao J, Wu D, Wang Z, Li P, Zhang W, Wu H, Feng N, Wang Z, Hua L, Wang X. 2011. [miR-143 decreases prostate cancer cells proliferation and migration and enhances their sensitivity to docetaxel through suppression of KRAS.](http://www.ncbi.nlm.nih.gov/pubmed/21197560) Mol Cell Biochem. 350:207-213.

Chen X, Guo X, Zhang H, Xiang Y, Chen J et al. 2009. [Role of miR-143 targeting KRAS in colorectal tumorigenesis.](http://www.ncbi.nlm.nih.gov/pubmed/19137007) Oncogene. 28:1385-92.

miR-145 Kent OA, Chivukula RR, Mullendore M, Wentzel EA, Feldmann G et al. 2010.[Repression of the miR-143/145 cluster by oncogenic Ras initiates a tumor-promoting feed-forward pathway.](http://www.ncbi.nlm.nih.gov/pubmed/21159816) Genes Dev. 24:2754-2759.

miR-181a Ota T, Doi K, Fujimoto T, Tanaka Y, Ogawa M et al. 2012. [KRAS up-regulates the expression of miR-181a, miR-200c and miR-210 in a three-dimensional-specific manner in DLD-1 colorectal cancer cells.](http://www.ncbi.nlm.nih.gov/pubmed/22641662) Anticancer Res. 32:2271-2275.

miR-181d Wang XF, Shi ZM, Wang XR, Cao L, Wang YY et al. 2012. [MiR-181d acts as a tumor suppressor in glioma by targeting K-ras and Bcl-2.](http://www.ncbi.nlm.nih.gov/pubmed/22207524) J Cancer Res Clin Oncol. 138:573-584.

miR-216b Deng M, Tang H, Zhou Y, Zhou M, Xiong W et al. 2011. [miR-216b suppresses tumor growth and invasion by targeting KRAS in nasopharyngeal carcinoma.](http://www.ncbi.nlm.nih.gov/pubmed/21878506) J Cell Sci. 124:2997-3005.

miR-217 Zhao WG, Yu SN, Lu ZH, Ma YH, Gu YM, Chen J. 2010.[The miR-217 microRNA functions as a potential tumor suppressor in pancreatic ductal adenocarcinoma by targeting KRAS.](http://www.ncbi.nlm.nih.gov/pubmed/20675343) Carcinogenesis. 31:1726-1733.

miR-622 Han Z, Yang Q, Liu B, Wu J, Li Y, Yang C, Jiang Y. 2012. [MicroRNA-622 functions as a tumor suppressor by targeting K-Ras and enhancing the anticarcinogenic effect of resveratrol.](http://www.ncbi.nlm.nih.gov/pubmed/22016468) Carcinogenesis. 33:131-139.

**2. IGF1R**

miR-7 [Zhao X](http://www.ncbi.nlm.nih.gov/pubmed?term=Zhao%20X%5BAuthor%5D&cauthor=true&cauthor_uid=22614005), [Dou W](http://www.ncbi.nlm.nih.gov/pubmed?term=Dou%20W%5BAuthor%5D&cauthor=true&cauthor_uid=22614005), [He L](http://www.ncbi.nlm.nih.gov/pubmed?term=He%20L%5BAuthor%5D&cauthor=true&cauthor_uid=22614005), [Liang S](http://www.ncbi.nlm.nih.gov/pubmed?term=Liang%20S%5BAuthor%5D&cauthor=true&cauthor_uid=22614005), [Tie J](http://www.ncbi.nlm.nih.gov/pubmed?term=Tie%20J%5BAuthor%5D&cauthor=true&cauthor_uid=22614005) et al. 2013. MicroRNA-7 functions as an anti-metastatic microRNA in gastric cancer by targeting insulin-like growth factor-1 receptor. [Oncogene.](http://www.ncbi.nlm.nih.gov/pubmed/22614005) 32:1363-1372.

miR-122 [Wang B](http://www.ncbi.nlm.nih.gov/pubmed?term=Wang%20B%5BAuthor%5D&cauthor=true&cauthor_uid=23056576), [Wang H](http://www.ncbi.nlm.nih.gov/pubmed?term=Wang%20H%5BAuthor%5D&cauthor=true&cauthor_uid=23056576), [Yang Z](http://www.ncbi.nlm.nih.gov/pubmed?term=Yang%20Z%5BAuthor%5D&cauthor=true&cauthor_uid=23056576). 2012. MiR-122 inhibits cell proliferation and tumorigenesis of breast cancer by targeting IGF1R. [PLoS One.](http://www.ncbi.nlm.nih.gov/pubmed/23056576) 7:e47053.

miR-139 [Shen K](http://www.ncbi.nlm.nih.gov/pubmed?term=Shen%20K%5BAuthor%5D&cauthor=true&cauthor_uid=22580051), [Liang Q](http://www.ncbi.nlm.nih.gov/pubmed?term=Liang%20Q%5BAuthor%5D&cauthor=true&cauthor_uid=22580051), [Xu K](http://www.ncbi.nlm.nih.gov/pubmed?term=Xu%20K%5BAuthor%5D&cauthor=true&cauthor_uid=22580051), [Cui D](http://www.ncbi.nlm.nih.gov/pubmed?term=Cui%20D%5BAuthor%5D&cauthor=true&cauthor_uid=22580051), [Jiang L](http://www.ncbi.nlm.nih.gov/pubmed?term=Jiang%20L%5BAuthor%5D&cauthor=true&cauthor_uid=22580051) et al. 2012. MiR-139 inhibits invasion and metastasis of colorectal cancer by targeting the type I insulin-like growth factor receptor. [Biochem Pharmacol.](http://www.ncbi.nlm.nih.gov/pubmed/22580051) 84:320-330.

miR-150* [Farhana L](http://www.ncbi.nlm.nih.gov/pubmed?term=Farhana%20L%5BAuthor%5D&cauthor=true&cauthor_uid=23675407), [Dawson MI](http://www.ncbi.nlm.nih.gov/pubmed?term=Dawson%20MI%5BAuthor%5D&cauthor=true&cauthor_uid=23675407), [Murshed F](http://www.ncbi.nlm.nih.gov/pubmed?term=Murshed%20F%5BAuthor%5D&cauthor=true&cauthor_uid=23675407), [Das JK](http://www.ncbi.nlm.nih.gov/pubmed?term=Das%20JK%5BAuthor%5D&cauthor=true&cauthor_uid=23675407), [Rishi AK](http://www.ncbi.nlm.nih.gov/pubmed?term=Rishi%20AK%5BAuthor%5D&cauthor=true&cauthor_uid=23675407), [Fontana JA](http://www.ncbi.nlm.nih.gov/pubmed?term=Fontana%20JA%5BAuthor%5D&cauthor=true&cauthor_uid=23675407). 2013. Upregulation of miR-150* and miR-630 Induces Apoptosis in Pancreatic Cancer Cells by Targeting IGF-1R. PLoS One. 8: e61015.

miR-153 [Song L](http://www.ncbi.nlm.nih.gov/pubmed?term=Song%20L%5BAuthor%5D&cauthor=true&cauthor_uid=23046980), [Duan P](http://www.ncbi.nlm.nih.gov/pubmed?term=Duan%20P%5BAuthor%5D&cauthor=true&cauthor_uid=23046980), [Guo P](http://www.ncbi.nlm.nih.gov/pubmed?term=Guo%20P%5BAuthor%5D&cauthor=true&cauthor_uid=23046980), [Li D](http://www.ncbi.nlm.nih.gov/pubmed?term=Li%20D%5BAuthor%5D&cauthor=true&cauthor_uid=23046980), [Li S](http://www.ncbi.nlm.nih.gov/pubmed?term=Li%20S%5BAuthor%5D&cauthor=true&cauthor_uid=23046980), [Xu Y](http://www.ncbi.nlm.nih.gov/pubmed?term=Xu%20Y%5BAuthor%5D&cauthor=true&cauthor_uid=23046980), [Zhou Q](http://www.ncbi.nlm.nih.gov/pubmed?term=Zhou%20Q%5BAuthor%5D&cauthor=true&cauthor_uid=23046980). 2012. Downregulation of miR-223 and miR-153 mediates mechanical stretch-stimulated proliferation of venous smooth muscle cells via activation of the insulin-like growth factor-1 receptor. [Arch Biochem Biophys.](http://www.ncbi.nlm.nih.gov/pubmed/23046980) 528:204-211.

miR-181b [Shi ZM](http://www.ncbi.nlm.nih.gov/pubmed?term=Shi%20ZM%5BAuthor%5D&cauthor=true&cauthor_uid=23431408), [Wang XF](http://www.ncbi.nlm.nih.gov/pubmed?term=Wang%20XF%5BAuthor%5D&cauthor=true&cauthor_uid=23431408), [Qian X](http://www.ncbi.nlm.nih.gov/pubmed?term=Qian%20X%5BAuthor%5D&cauthor=true&cauthor_uid=23431408), [Tao T](http://www.ncbi.nlm.nih.gov/pubmed?term=Tao%20T%5BAuthor%5D&cauthor=true&cauthor_uid=23431408), [Wang L](http://www.ncbi.nlm.nih.gov/pubmed?term=Wang%20L%5BAuthor%5D&cauthor=true&cauthor_uid=23431408) et al. 2013. MiRNA-181b suppresses IGF-1R and functions as a tumor suppressor gene in gliomas. [RNA.](http://www.ncbi.nlm.nih.gov/pubmed/23431408/) 19:552-560.

miR-223 [Jia CY](http://www.ncbi.nlm.nih.gov/pubmed?term=Jia%20CY%5BAuthor%5D&cauthor=true&cauthor_uid=22073238), [Li HH](http://www.ncbi.nlm.nih.gov/pubmed?term=Li%20HH%5BAuthor%5D&cauthor=true&cauthor_uid=22073238), [Zhu XC](http://www.ncbi.nlm.nih.gov/pubmed?term=Zhu%20XC%5BAuthor%5D&cauthor=true&cauthor_uid=22073238), [Dong YW](http://www.ncbi.nlm.nih.gov/pubmed?term=Dong%20YW%5BAuthor%5D&cauthor=true&cauthor_uid=22073238), [Fu D](http://www.ncbi.nlm.nih.gov/pubmed?term=Fu%20D%5BAuthor%5D&cauthor=true&cauthor_uid=22073238) et al. 2011. MiR-223 suppresses cell proliferation by targeting IGF-1R. PLoS One 6:e27008.

miR-376a [Zehavi L](http://www.ncbi.nlm.nih.gov/pubmed?term=Zehavi%20L%5BAuthor%5D&cauthor=true&cauthor_uid=22747855), [Avraham R](http://www.ncbi.nlm.nih.gov/pubmed?term=Avraham%20R%5BAuthor%5D&cauthor=true&cauthor_uid=22747855), [Barzilai A](http://www.ncbi.nlm.nih.gov/pubmed?term=Barzilai%20A%5BAuthor%5D&cauthor=true&cauthor_uid=22747855), [Bar-Ilan D](http://www.ncbi.nlm.nih.gov/pubmed?term=Bar-Ilan%20D%5BAuthor%5D&cauthor=true&cauthor_uid=22747855), [Navon R](http://www.ncbi.nlm.nih.gov/pubmed?term=Navon%20R%5BAuthor%5D&cauthor=true&cauthor_uid=22747855) et al.. 2012. Silencing of a large microRNA cluster on human chromosome 14q32 in melanoma: biological effects of mir-376a and mir-376c on insulin growth factor 1 receptor. Molecular Cancer 11:44.

mir-376c [Zehavi L](http://www.ncbi.nlm.nih.gov/pubmed?term=Zehavi%20L%5BAuthor%5D&cauthor=true&cauthor_uid=22747855), [Avraham R](http://www.ncbi.nlm.nih.gov/pubmed?term=Avraham%20R%5BAuthor%5D&cauthor=true&cauthor_uid=22747855), [Barzilai A](http://www.ncbi.nlm.nih.gov/pubmed?term=Barzilai%20A%5BAuthor%5D&cauthor=true&cauthor_uid=22747855), [Bar-Ilan D](http://www.ncbi.nlm.nih.gov/pubmed?term=Bar-Ilan%20D%5BAuthor%5D&cauthor=true&cauthor_uid=22747855), [Navon R](http://www.ncbi.nlm.nih.gov/pubmed?term=Navon%20R%5BAuthor%5D&cauthor=true&cauthor_uid=22747855) et al. 2012. Silencing of a large microRNA cluster on human chromosome 14q32 in melanoma: biological effects of mir-376a and mir-376c on insulin growth factor 1 receptor. Molecular Cancer 11:44.

miR-383 [He Z](http://www.ncbi.nlm.nih.gov/pubmed?term=He%20Z%5BAuthor%5D&cauthor=true&cauthor_uid=23564324), [Cen D](http://www.ncbi.nlm.nih.gov/pubmed?term=Cen%20D%5BAuthor%5D&cauthor=true&cauthor_uid=23564324), [Luo X](http://www.ncbi.nlm.nih.gov/pubmed?term=Luo%20X%5BAuthor%5D&cauthor=true&cauthor_uid=23564324), [Li D](http://www.ncbi.nlm.nih.gov/pubmed?term=Li%20D%5BAuthor%5D&cauthor=true&cauthor_uid=23564324), [Li P](http://www.ncbi.nlm.nih.gov/pubmed?term=Li%20P%5BAuthor%5D&cauthor=true&cauthor_uid=23564324), [Liang L](http://www.ncbi.nlm.nih.gov/pubmed?term=Liang%20L%5BAuthor%5D&cauthor=true&cauthor_uid=23564324), [Meng Z](http://www.ncbi.nlm.nih.gov/pubmed?term=Meng%20Z%5BAuthor%5D&cauthor=true&cauthor_uid=23564324). 2013. Downregulation of miR-383 promotes glioma cell invasion by targeting insulin-like growth factor 1 receptor. [Med Oncol.](http://www.ncbi.nlm.nih.gov/pubmed/23564324) 30:557.

miR-493 [Okamoto K](http://www.ncbi.nlm.nih.gov/pubmed?term=Okamoto%20K%5BAuthor%5D&cauthor=true&cauthor_uid=22373578), [Ishiguro T](http://www.ncbi.nlm.nih.gov/pubmed?term=Ishiguro%20T%5BAuthor%5D&cauthor=true&cauthor_uid=22373578), [Midorikawa Y](http://www.ncbi.nlm.nih.gov/pubmed?term=Midorikawa%20Y%5BAuthor%5D&cauthor=true&cauthor_uid=22373578), [Ohata H](http://www.ncbi.nlm.nih.gov/pubmed?term=Ohata%20H%5BAuthor%5D&cauthor=true&cauthor_uid=22373578), [Izumiya M](http://www.ncbi.nlm.nih.gov/pubmed?term=Izumiya%20M%5BAuthor%5D&cauthor=true&cauthor_uid=22373578) et al.2012. miR-493 induction during carcinogenesis blocks metastatic settlement of colon cancer cells in liver. [EMBO J.](http://www.ncbi.nlm.nih.gov/pubmed/22373578) 31:1752-1763.

miR-630 [Farhana L](http://www.ncbi.nlm.nih.gov/pubmed?term=Farhana%20L%5BAuthor%5D&cauthor=true&cauthor_uid=23675407), [Dawson MI](http://www.ncbi.nlm.nih.gov/pubmed?term=Dawson%20MI%5BAuthor%5D&cauthor=true&cauthor_uid=23675407), [Murshed F](http://www.ncbi.nlm.nih.gov/pubmed?term=Murshed%20F%5BAuthor%5D&cauthor=true&cauthor_uid=23675407), [Das JK](http://www.ncbi.nlm.nih.gov/pubmed?term=Das%20JK%5BAuthor%5D&cauthor=true&cauthor_uid=23675407), [Rishi AK](http://www.ncbi.nlm.nih.gov/pubmed?term=Rishi%20AK%5BAuthor%5D&cauthor=true&cauthor_uid=23675407), [Fontana JA](http://www.ncbi.nlm.nih.gov/pubmed?term=Fontana%20JA%5BAuthor%5D&cauthor=true&cauthor_uid=23675407). 2013. Upregulation of miR-150* and miR-630 Induces Apoptosis in Pancreatic Cancer Cells by Targeting IGF-1R. PLoS One. 8: e61015.

__________________________________________________________________________________________________________
